# Evaluating quality neonatal care, call Centre service, tele-health and community engagement in reducing newborn morbidity and mortality in Bungoma county, Kenya

**DOI:** 10.1186/s12913-018-3293-5

**Published:** 2018-06-25

**Authors:** Jesse Gitaka, Alice Natecho, Humphrey M. Mwambeo, Daniel Maina Gatungu, David Githanga, Timothy Abuya

**Affiliations:** 1 Research and Innovation Centre, Mount Kenya University, P.O. Box 342-01000, Thika, Kenya; 2 Fountain Africa Trust, P.O. Box 1632-50205, Webuye, Bungoma, Kenya; 3 Kenya Paediatric Association, P.O. Box 45821-00100, GPO, Nairobi, Kenya; 4 Population Council, Kenya P. O. Box, Nairobi, 17643-00500 Kenya

**Keywords:** Neonatal mortality, Community sensitisation, Newborn units, Telehealth, Call centre

## Abstract

**Background:**

Neonatal mortality is a major health burden in Bungoma County with the rate estimated at 31 per 1000 live births and is above the national average of 22 per 1000. Nonetheless, out of the nine sub county hospitals, only two are fairly equipped with necessary infrastructure and skilled personnel to manage neonatal complications such as prematurity, neonatal sepsis, neonatal jaundice, birth asphyxia and respiratory distress syndrome. Additionally, with more than 50% of neonates delivered without skilled attendance, in below par hygiene environments such as home and on the roadsides, with non-existent community based referral system, the situation is made worse. The study aims to evaluate the progress made by an intervention “Collaborative Newborn Support Project” geared towards reducing neonatal mortality rate by 30% between October 2015 and December 2018 in Bungoma County, Kenya.

**Methods/Design:**

This intervention will take a quasi-experimental design approach with experimental and control sites. The project will involve pre- and post-intervention data collection with comparison group to assess intervention effects. The primary outcome will be the percentage reduction of neonatal mortality in Bungoma County. Secondary outcomes include; a) Percentage of mothers or care givers able to identify at least three danger signs in neonates in the project area, b) Proportion of neonates with complications referred to specialized neonatal centers, through the call center, c) Percentage of health providers in neonatal care units who adhere to expected neonatal standards of care (rapid and complete application of standard protocols), d) Percentage increase in neonates with severe complications in the specialized neonatal units and e) Percentage of neonates who stay in neonatal care units beyond 5 days.

**Discussion:**

We outline implementation details of the ongoing ‘Collaborative Newborn Support Project’ in Bungoma County, Kenya. This includes strategies in the operations of the telehealth platform, call centre service, community engagement and measuring of the outputs and outcomes. The funding and ethical approvals have been obtained and the study commenced.

**Trial registration:**

PACTR201712002802638 Retrospectively registered on 5th December 2017 at Pan African Clinical Trials Registry.

**Electronic supplementary material:**

The online version of this article (10.1186/s12913-018-3293-5) contains supplementary material, which is available to authorized users.

## Background

Approximately 2.7 million new-borns die each year accounting to 43% of deaths of children under 5 years [1]. Maternal and new-born deaths were the focus of millennium development goals 4 and 5 which were not met by 2015 and are currently captured in the sustainable development goals [2, 3]. Kenya has a big neonatal mortality burden currently at 22 out of 1000 live births with Bungoma County at 31 per 1000 live births [4]. Major contributors to the morbidity and mortality challenge include poor new-born health coverage, low levels of perinatal care [5], adverse cultural practices including unskilled home deliveries, poor cord care comprising application of cow dung, lizard excreta, herbs and mud to ‘facilitate’ cord healing and early weaning. Additionally, the continuum of care from antenatal care to delivery and postnatal care and new-born health, is highly disjointed in most rural districts due to inadequate capacities (infrastructural, personnel and supplies) and strategies worsening the situation [6–8].

A key pillar in supporting strategies to mitigate new-born morbidity and mortality is improvement of quality of care. Even though there is no clear definition of new-born quality of care, important elements include; skilled care during pregnancy and delivery encompassing skilled birth attendance, emergency obstetric care for maternal and new-born complications and post-natal care for mothers and babies [9, 10]. In defining quality of care in regards to maternal health, Hulton et al.*,* [11] point to effective and timely access to services. The WHO has published a comprehensive quality of care guideline titled ‘Standards for improving quality of maternal and newborn care in health facilities’ that aims to set the bar especially for the developing world [12]. Kenya has developed its own quality strategy called ‘The Kenya Quality Health Model’ that strives to guide different sectors of health provision including maternal and newborn on attaining International Standards Organisation certification. Nonetheless, the model does not cover process implementation but provides tools for continuous quality improvement, the Plan-Do –Check- Act cycle et cetera [13]. The quality of care guidelines will of necessity be live to the different emerging strategies in increasing access to quality care especially in peripheral or poor resource settings factoring diverse metrics in their evaluation [14]. These include mobile and telehealth applications, call centre services and other technologies.

The collaborative new-born support was conceived to address the unacceptably high new-born morbidity and mortality rates in Bungoma County with the purpose of enhancing survival of neonates, improve quality of care and accessibility of new-born care services in the nine sub-counties of Bungoma.

### Anticipated impact

The project will contribute towards the two outcomes with activities linking as follows; 1. Increased access to and utilisation of quality neonatal health services since new-born special care units in 7 sub county hospitals will be set-up, introducing these services to the sub counties, establishing a mobile phone based follow up system for neonates will empower mothers to make timely health seeking decisions and avert complications. The community sensitization sessions will inform the members and stakeholders on availability of the services stimulating demand.

Secondly, the project will contribute to health system management strengthening to deliver quality neonatal health services because 90 medical staff will be trained, mentored and supported through a tele-health platform by paediatricians from Kenya Paediatric Association and Mount Kenya University. It is anticipated that this strategy will build the healthcare workers confidence in neonatal care and create a pool of trainers in the rural facilities. Moreover, repackaging and distributing 1000 copies of best practice neonatal clinical guidelines to facilities in the county will provide quick reference. A community referral system for neonatal health services will be enhanced working with community based midwives. Besides, it is anticipated that mothers and particularly with pre-natal challenges will choose to deliver in these facilities for better neonatal outcomes, raising skilled attendant deliveries.

### Hypothesis

1. Quality neonatal care, call centre service, tele-health and community engagement will reduce new-born morbidity and mortality in Bungoma county, Kenya

### Study questions

1. To what extent does increase in community knowledge on neonatal complications and availability of quality services affect mothers/new born caregivers utilization of hospital services in Bungoma County?
2. Will improved quality of neonatal care lead to an increase in utilization of new born care services in Bungoma County?
3. Does improved information flow amongst health practitioners result in better services for neonates in Bungoma County?

## Methods

### Study setting

Bungoma County (coordinates 0.8479° N, 34.7020° E), Western Kenya has a population of approximately 1.7 m and an area of 2069 km^2^. The County has an urbanization rate of 21.7%, literacy levels of 60.5% with 87.6% of residents between the ages of 15–18 attending primary school. It has a poverty rate of 52.9% and access to electricity access of 4.5% [4, 15]. The main ethnic groups in the county are the Bukusu and Sabaoti sub-tribes of Luhya and Kalenjin respectively.

The main economic activities include: Agriculture, manufacturing and retail services. Agriculture is the backbone of Bungoma County and most families rely on crop production and animal rearing. The main crops include maize, beans, finger millet, sweet potatoes, bananas, Irish potatoes and assorted vegetables. These are grown primarilly for subsistence with the excess sold to meet other family needs. On the other hand, the main cash crops include sugar cane, cotton, palm oil, coffee, sun flower and tobacco. Most families integrate livestock production with farming. The main livestock kept include cattle, sheep, goats, donkeys, pigs, poultry and bees. Most of this is on a small scale but some farmers also produce milk and poultry products for commercial use. Milk and sugarcane farmers sell their produce mainly through cooperative societies.

The County is comprised of 6 constituencies: Kimilili, Webuye East, Webuye West, Sirisia, Kanduyi, Bumula and Mt. Elgon. This guided the selection of project intervention sites. These sites are in the following facilities; Bungoma County referral hospital, Webuye Sub-County hospital, Kimilili, Sirisia, Bumula, Naitiri, Mt. Elgon, Sinoko and Chwele.

### Study design

This intervention will take a quasi-experimental design approach with an experimental and control site. The project will involve pre- and post-intervention data collection with one comparison group to assess intervention effects.

The study will be conducted at level 4 and 5 hospitals, each hospital having high volume of clients and covering a sub-county, all with similar maternal, newborn and child health indicators. Five hospitals will be the implementation sites while 4 hospitals will be control sites in a step wedge approach. The implementation sites will be selected purposely. The control sites will receive the full intervention after 1 year when the first set of comparison with the intervention site has been completed.

The 5 experimental site hospitals will receive a set of interventions which include: refurbishing of new born units, installation of equipment such as incubators, respirators, Ambu bags, and radiators. Establishment of a tele-health platform to enable free flow of information amongst clinicians and obstetricians, establishment of a call center for follow up of patients, training of health care providers in neonatology, and creating awareness amongst the community will be key cross cutting interventions, implying they will benefit the intervention site as well as control site. The control sites will not receive the main intervention benefits (equipped specialized newborn units) until after 1 year of implementation in the experimental sites.

The project will undertake a baseline, mid-term and end-line surveys. The step wedge design which allows for the measurement of process, outcome and impact indicators within each site (at different time points) will be used. The design also allows a comparison between different combinations of the intervention components.

### Primary outcome measure

Percentage reduction in newborn deaths in Bungoma County between October 2015 and December 2018. This will be measured by comparing the number of newborn deaths at the nine project facilities (in the period of implementation), with the number of deaths at the same facilities in the similar period before the intervention; converted to percentage and aggregated by facility.

### Secondary outcome measures

1. Percentage of mothers or care givers able to identify at least three danger signs in neonates in the project area; **Numerator**: Number of mothers/ care givers who can identify at least three danger signs in neonates in the project area**. Denominator**: Sampled mothers/care givers in selected community areas at baseline and end line.
2. Proportion of neonates with complications referred to specialized neonatal units, through the call center; **Numerator**: Number of neonates with complications referred to specialized neonatal centers, through the call center. **Denominator**: Total number of neonates reached through the call center.
3. Percentage of health providers in neonatal care units who adhere to expected neonatal standards of care (rapid and complete application of standard protocols); **Numerator**: Number of health workers in neonatal care who adhere to expected standards of neonatal care in the nine selected facilities. **Denominator**: Total number of health workers in neonatal care in the selected facilities.
4. Percentage increase in neonates with severe complications in the specialized neonatal units; Percentage change in the number of admissions of neonates classified as having severe complications (using the 15-score index) over the project period.
5. Percentage of neonates who stay in NBU beyond 5 days; **Numerator**: Number of neonates who stay beyond 5 days. **Denominator**: Total number of neonates admitted in the 9 specialized neonatal units

### Sources of data collection

#### Quantitative data

- Population within project sites
- Routine data from the selected sites(facilities)
- Baseline survey

#### Qualitative

Tools

- Baseline survey
- Observations
- Project information

### Control group – Minimizing bias and contamination

To minimize bias and contamination, the intervention sites will be selected purposely from the nine county and sub- county hospitals. The sites selected will include the two facilities that have some level of neonatal facilities to ensure that the intervention sites and the control sites have distinct features separating the two.

We will measure the utilization of services in the selected facilities at baseline, mid-term and end-line levels.

To determine utilization of neonatal services, we will collect data directly from the source through a data abstraction form in the nine health facilities throughout the project period. Further, the community health workers and volunteers will also provide information of the number of cases they refer to specific facilities. Cases that fail to arrive at the health facilities will also be captured and the data from the facilities and the community health workers will be cleaned, cross checked and analyzed to provide required results for utilization of neonatal services.

Quality of care will be determined through observations of service provision by clinicians, as well as checking use of neonatal equipment and the severity of conditions managed by the health providers in the units.

### Sample size determination

#### Selection of community members (newborn mothers)

Bungoma County, the area targeted by community sensitization will be source area for target newborn caregivers/newborn mothers who will be simple randomly selected to participate in the survey. Statistical methods for determining sample size will be employed to ensure the correct sample size is picked to avoid biases and ensure the outcomes are valid. There are approximately 29,845 births in Bungoma County per annum [16]. This number will be split twice for control and intervention group. The study will allow a margin of error of 5% at confidence level of 95% to obtain the correct sample size for the study. It is also expected that at least one of the variables of interest (e.g., knowledge of 3 danger signs in neonates) will be 50% among the respondents. Using the formula:

$$ x=Z{\left({}^c{/}_{100}\right)}^2r\left(100-r\right) $$

$$ n{=}^{N\ x}{{/_{\Big(\left(N-1\right)E}}^2}_{+x\Big)} $$

$$ E=\mathrm{Sqrt}\left[{}^{\left(N-n\right)x}{/}_{n\left(N-1\right)}\right] $$

where *N* is the population size, *r* is the fraction of responses that we are interested in, and *Z*(*c*/100) is the critical value for the confidence level *c*, we get 375 as our ideal sample size in the control and intervention areas (i.e., four data collectors in each area conducting 8 surveys daily for 12 working days each). See Additional file 1.

**Table 1:** Outcomes and objectives

Outcomes	• Increased access and utilization of quality neonatal care services. • Health system management strengthened to deliver quality neonatal health services.
Objectives	i) To setup 7 differentiated neonatal special care units in 7 sub-county hospitals in Bungoma. ii) To train 90 selected health workers (nurses and clinicians) based in 9 sub county hospitals in neonatal care to be able to diagnose and manage common new-born problems and make timely referral when appropriate. iii) Establishing a tele-health platform that will ease diagnostic and new-born clinical care consultant support iv) Re-packaging and distribution of best-practice clinical guidelines into user friendly formats. v) Develop a mobile phone based follow up system for all new-borns for early detection of neonatal problems and initiation of appropriate action.

#### Selection of hospitals for the study

There are 177 health facilities in Bungoma County of which 12 are hospitals and the rest are health centers, dispensaries and clinics. In this study, nine high volume health facilities will be selected to participate in the study. Five Facilities will be intervention sites while four health facilities will be the control sites. The intervention sites will be selected purposely from the nine project facilities.

#### Selection of participants for the clinical observations of neonatal services

The sample size calculation is based on net change in the proportion of neonates who present with complications in health facilities. From the County HMIS data, it is estimated that a significant proportion of babies born with complications are missed out or only a few of them present to health facilities. In Bungoma County, currently only 21% present in health facilities. Through the Newborn collaborative support project, we seek to increase this by 50%, (21 to 32%). Given that births are 29,845 [16] and current situation is 21% presenting in health facilities we get a population of 6268 neonates. This means we get a sample size of 125 neonates for intervention and 125 for control group. Data collection tools such as questionnaires will be administered randomly amongst the selected cases. See Additional file 2.

#### Selection of health care workers

Ninety health care providers who give informed consent to participate in the study and meet the requirement of the selection criteria will be interviewed through a questionnaire to determine their knowledge and skills levels in neonatal care. The health care providers have already been trained in their respective areas but may be deficient in neonatal care. It is approximated that their knowledge on neonatology is at 65% and this needs to be increased up to about 90%. See Additional file 3.

#### Participants for in-depth interviews

In- depth interviews will be conducted with key informants in the selected project sites (See Additional file 4). They include the following:

1 – County Director of health services

1 – Director of nursing services in the county

1 – County RH coordinator

1 –Pediatrician in the county

9 – Medical Superintendents/Facility –in- charges – one in each Hospital

9- Nurses in – charge on maternity – one in each Facility

9- Staff in- charge of new born units

## 9 – In- charge of PNC services

### Limitation of sampling technique selected

The simple random sampling technique that will be employed in this study will require a lot of time to implement and is quite tedious. This implies that more resources shall be needed for the study and the implementing team will have to be robust enough to withstand the rigorous exercise.

### Quantitative data

- Questionnaires –

i. This will be administered to health workers to check their knowledge, attitudes and practices in neonatal care.
ii. Community (mothers of newborns/caregivers) to assess effect of information on their knowledge of danger signs and utilization of neonatal services.

- Check list – Will consist of a list of equipment available in the new born care unit, their condition, and frequency of use
- Data abstraction form –This will be developed to collect routine health facility records in the neonatal unit to ensure completeness and accuracy in data generated. The trained nurses and clinical officers will ensure correct data entry which will be monitored periodically by the hospital facility in conjunction with the project. The trained staff will also mentor other staff in the facility (OJT) to also develop competency and correct data recording and entry.
- Client exit interviews –will be administered to clients from new born unit to capture the perception of clients on quality of care provided, and the outcome of the service.

### Qualitative

#### Tools

- Provider observed interviews – observation of neonatal care services will be conducted to determine the quality of care provided by clinicians
- In-depth Interviews- for key informants to check on policies and practices surrounding new born health services. It will include policy of deployment of personnel to various units, performance assessment of personnel, availability of drugs and supplies.
- 15 score -Severity of illness of the babies admitted in specialized neonatal units in the project sites.

### Operationalization of the telehealth platform

#### Brief description

The telehealth platform will provide a virtual consultation support environment for the clinicians in the special care units and pediatricians on call. Additionally, it will be used for continuous medical education and mentorship of the clinicians.

#### Rationale

1. The project will establish 7 new special care units and enhance capacity in 2. Following the 3 months module based competency based neonatology course for 90 health care workers in these units, it will be important to provide medical consultation support by pediatricians. This will facilitate timely clinical decision making including and not limited to; judicious use of medicines such as antibiotics, change of treatment, fluids administration, nutritional calculations, immunization decisions, evolution of diagnosis, need for referral and follow up strategies. This virtual support will build the clinicians confidence in clinical management of newborns, knowing that they can ask and get help at any time of the day, all week, ultimately improving outcomes.
2. A major challenge for clinicians working in peripheral, rural areas in Kenya, has been keeping in touch with best care practices due to inability to access this information, lack of continuous education on how to apply the updates on best care and failure of follow up on adoption of best care practices. The telehealth platform will provide virtual best care practices updates service regularly and interactively. Pediatricians and the special units’ clinicians will be able to discuss on implementation of the updates, challenges they face and lessons learnt. This will promote free flow of information between the consultants and the clinicians, promoting best care practices and newborn outcomes.
3. The telehealth platform will enable data collection on clinical challenges and gaps that will inform project evaluation, areas of emphasis in the continuous medical education and enhanced efficiencies in other processes such as call center follow up and referral systems.

#### Operation

The telehealth platform is a virtual support system. Clinicians and pediatricians will be supplied with smart phones with a telehealth application and internet accessibility. For consultations, the special care units’ clinicians will be able to post patient data such as clinical history, examination findings, laboratory results, imaging data, treatment and evolution of clinical status. The pediatrician on call will then provide the necessary professional advice. The consultation will be tracked to conclusion and archived for analysis. Servers will be housed at Mount Kenya University, main campus, Thika, Kenya and the IT support services provided by developer and the department of IT. Best care practices will be posted by project lead and pediatrician regularly.

#### Inputs

1. Smart phones
2. Computers
3. Telehealth standard operating procedures
4. Telehealth software
5. Internet charges
6. Servers and maintenance

#### Outputs

1. Telehealth consultation data
2. Number of beneficiaries and outcomes
3. Postings of best care practice guidelines
4. Discussion forum data
5. Lessons learnt

### Beneficiaries

Newborns and their mothers.

### Operationalization of the call center service

#### Brief description

The call center service will link between the special care units and the community based midwifes and mothers of newborns. It will provide information to mothers in supporting their ability to make decisions on wellness of the newborns in the community and health seeking decisions such as urgency of taking their babies to hospital and which facility will be most appropriate. Besides, it will be used as a follow up strategy after discharge. Additionally, the service will post messages promoting newborn health for use by mothers and women on antenatal clinic follow up. Ultimately, the call center will facilitate development of a community referral system for newborns, improve mothers of newborns health knowledge, timely decision making and outcomes.

#### Rationale

1. More than 50% of neonates in Bungoma County are delivered without skilled attendance, in below par hygiene environments such as home and on the roadsides. This exposes the newborns to risk of infection among other complications. Often, these newborns fall ill and delay in seeking health services worsens outcomes. The call centre service, aims to streamline community based referral system, whereby, community based midwifes will report all deliveries in their locations to the call centre and the newborn mothers will be contacted and enrolled for follow up and provided with information to be able to make timely decisions for health seeking besides being sensitised on the availability of the special care units services near them.
2. Newborn follow up after discharge is a critical component of care in sustaining wellness beyond the first month of life. A significant number of mothers may not be able to detect early symptoms of newborn illness such as sepsis or jaundice. The call centre service will actively prompt mothers on follow up after discharge to check out on symptoms and advise accordingly.
3. Women on antenatal care follow up need information on how to nurture their newborns after delivery. Nonetheless, it has been noted that ante natal clinic attendance varies widely with drastic falls between second, third and fourth visits. Besides, since most of the health education sessions are held in the morning, mothers coming late do not attend and lose out on the education. This service will augment the ante natal care clinics in providing this information using calls and text messaging in addition to encouraging clinic attendance. The high mobile telephony penetration in the county makes this service feasible.
4. The call centre service will provide a link between the community and the telehealth platform by providing community derived data that will inform project monitoring, strategies to enhance efficiencies in processes such as referral systems, community based midwifes engagements and newborn follow up outcomes.

#### Operation-how the call center service will work

1. In order to support newborns’ mothers on checking on their babies’ health through follow up, we plan to use the following strategies:

For mothers who are able to read and write an sms, the call center attendant will send out an sms 3 times a week in the mothers best language of comprehension (e.g. Kiswahili, Kibukusu, Kisabaot etc.) prompting them to check and feedback on the following:

- Ability of the newborn to breastfeed
- Symptoms of dehydration
- Irritability
- Breathing- ease or difficulty
- Hotness or ‘coldness’ of body

2. For mothers who are not able to read or write an sms, the call center attendant will make a call 3 times a week in the mother’s best language of comprehension prompting on the above a-e.
3. In case responses following the above enquiries are not satisfactory (or point to a baby having a health problem) they shall be advised on immediate measures to take and referred to the special care units.
4. Mothers will be able to initiate the sms or call in case they need any help or information.
5. Immunization information will also be provided.
6. Expectant mothers will be called or texted with messages promoting knowledge on newborn health such as exclusive breastfeeding, immunization, hygiene and danger symptoms.

#### Inputs

1. Call center manual of operation
2. Calling and texting equipment; Smart phones and computers
3. Office furniture
4. Calling credit
5. Call center attendants

#### Outputs

1. Number of calls
2. Number of referrals
3. Community events based surveillance data fed into the telehealth platform
4. Number of newborns followed up.
5. Number of expectant mothers followed up and messaged

#### Beneficiaries

1. Mothers of newborns
2. Newborns in the community
3. Expectant women

#### Interphase of telehealth platform and call center

**Fig. 1:**
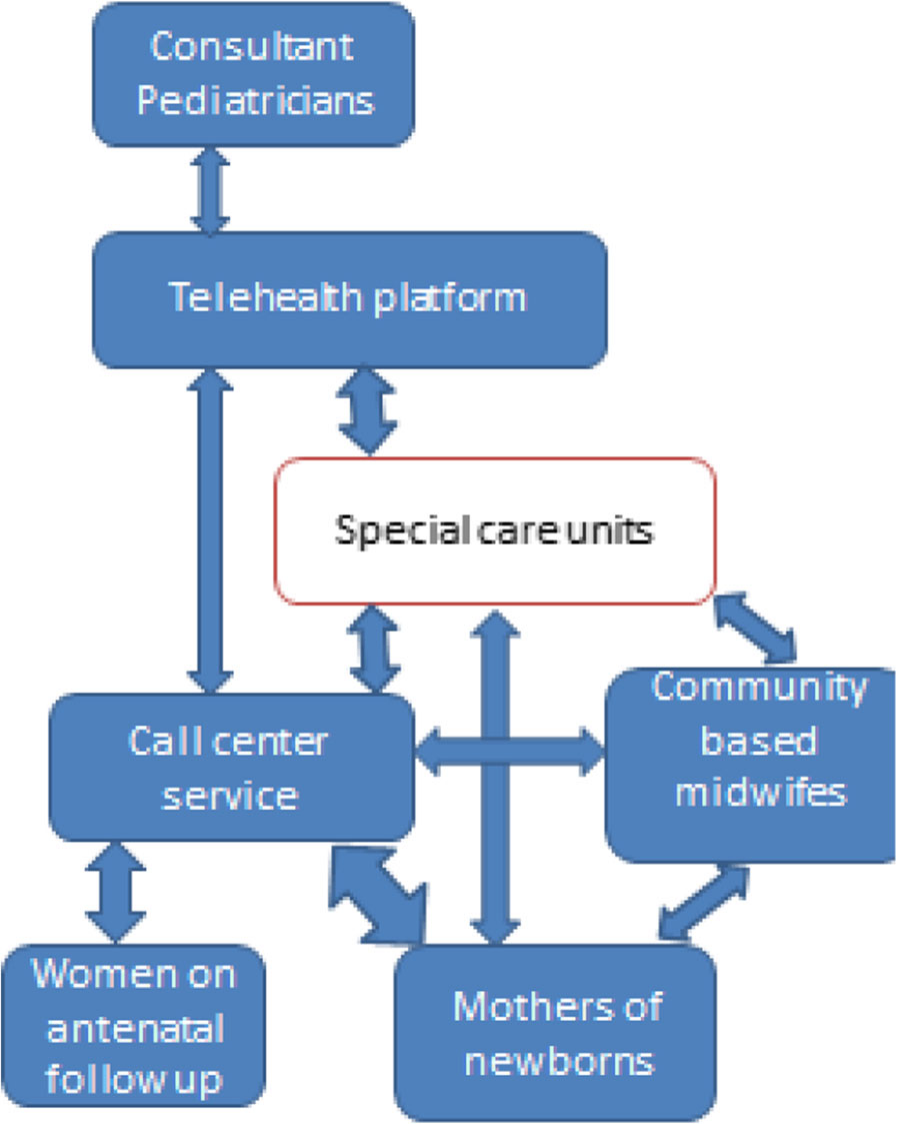
Interphase of the telehealth platform and the call centre service

The two innovations, telehealth and call center are key components in the success of the project. These will interphase as follows;

1. The call center will promote community referral system linkage with the community based midwifes and the special care units, promoting utilization of the units. The telehealth platform will enhance the capacity of the clinicians in these units raising standards of care, user satisfaction and ultimately improving neonatal health outcomes.
2. The call center will focus on follow up of the neonates born within the county health care system and without. The follow up data will be uploaded into the telehealth platform allowing the project pediatricians and clinicians understand the situation of the newborns in the community, respond to queries arising during follow up and streamline care and discharge processes in the special care units. See Fig. 1.

#### Privacy, informed consent and confidentiality

Every person’s privacy shall be respected and where they are required to provide information they shall be informed of the purpose for which the information is required, informed of their right to withdraw from the interview at any stage and time, and that information will not be divulged to third parties. Further, the subjects will be informed of the fact that their right to medical attention and other natural privileges will not be affected whatsoever by their agreeing to participate or refusing to participate in the interviews to be conducted. This information will be shared to target subjects in a language understood and where possible in writing.

Subjects will also be informed of the fact that all information collected for the purpose of the study shall solely be used as such and subsequently destroyed after about 3 years following completion of the study. No identifiers will be used such that information collected shall not be linked to the persons who shall have provided such information.

#### Control group

The study ensures that the control group will also receive the full intervention towards the end of the project period so that they do not remain disadvantaged. Initially they shall benefit from community sensitization, tele health platform and call center. After a period of 1 year, the control group will be ready for the full intervention which will include establishment of functional specialized neonatal units.

#### Study assumption

The main assumption in this study is that the intervention and control sites have similar characteristics. Since the facilities are under different Health Facility Management Teams, and health priorities, and decisions made by the HFMT are dependent on the catchment community of each facility, there is a possibility that the intervention and control sites are not necessarily comparable. However, since the sites have low PNC coverage and similar challenges affecting access and utilization of neonatal health services, it is assumed that the differences will not affect the outcome of the study.

## Discussion

The innovation has four components supporting each other as follows;

- Operation of telehealth platform which is an innovative tool that supports teleconsultations between professional paediatricians and the clinical offices at different health facilities. The telehealth platform essentially provides solution to the challenge of inadequate professionals in the target area which currently has a single paeditrician serving a total population of approximately 1.7 Million people. It enables realtime consultations between the clinical officers and the paeditricians. In addition, it enables professionals in distant locatilities bridge the gap by providing required services. There are 5 professionals from Nairobi (a distance of approximately 500 Kilometers) serving the population in addition to the one resident professional. It works by having the clinical officers take images of newborns under stress and share the same alongside clinical notes with professionals for advice in realtime.
- Call centre enables the call centre attendants who are trained nurses to consult with mothers of newborns and offer advice aimed at shortening decision making to seek care. This handles the challenge of delayed decisions to seek medical care. The mothers will call the call centre and present any challenges they face with their newborns with an aim of getting advise to handle the danger signs that they may have noted with their newborns. This enables them to make decisions to seek required medical care on time.
- Community engagement involves sensitizing the community members especially mothers of newborns of improved facilities within their locality. In addition, the community members are trained on how to identify danger signs in newborns and take remedial action in good time. This approach reduces the delay in seeking care for the newborns when required. When the community is sensitized, it becomes proactive and make relatively good decisions concerning the health of their newborns.
- Measuring results (outputs and outcomes) will involve undertaking a baseline survey to establish benchmarks that will be used to gauge the success of the intervention. This survey will involve the community within the target area, clinicians in selected health facilities and administrators of the health facilities including the County government officials who are legally incharge of the health facilities in the region. Outputs and outcomes will be established through internal and external evaluations which will be conducted at midterm and endterm of the project intervention (see Table 1). These will consider the benchmarks established at baseline to establish change in the situation beased on the intervention. This evaluation will be exhaustive enough and objective to ensure that only results that can be attributed to the intervention will be included since there are many other efforts towards improving the helthcare of newborns in the target area.

## Additional files

Additional file 1: House Hold Interview Tool for Mothers and Care Takers. (DOCX 67 kb)

Additional file 2: Tool for Service Provider - Client Observation. (DOCX 103 kb)

Additional file 3: Tool for health provider’s interview. (DOCX 67 kb)

Additional file 4: Guide for key informant interviews for health managers. (DOCX 56 kb)

## Abbreviations

CICF: County Innovation Challenge Fund
HFMT: Health Facility Management Teams
HMIS: Health Management Information Systems
KDHS: Kenya Demographic and Health Survey
KNBS: Kenya National Bureau of Statistics
OJT: On job training
PNC: Post-Natal Care
WHO: World Health Organisation
