# Supplementary material for: Evaluating quality neonatal care, call Centre service, tele-health and community engagement in reducing newborn morbidity and mortality in Bungoma county, Kenya
Source: BMC Health Serv Res. 2018 Jun 25;18:493. doi: 10.1186/s12913-018-3293-5 (PMC6019716; doi:10.1186/s12913-018-3293-5)
Supplement: Supplementary file 1 — House Hold Interview Tool for Mothers and Care Takers. (DOCX 67 kb) [file 12913_2018_3293_MOESM1_ESM.docx]

**QUESTIONNAIRE NUMBER: (______________)**

**House Hold Interview Tool for Mothers And Care Takers**

**Collaborative Newborn Support Project**

| **IDENTIFICATION INFORMATION** | |
| --- | --- |
| Name of County: |  |
| Name of Sub County: |  |
| Name of Division: |  |
| Name of Location: |  |
| Name of Sub-Location: |  |
| Name of Assistant Chief: |  |
| Name of Village |  |
| Name of Headman/Village Elder |  |
| Name of nearest school |  |
| Name of other landmark (e.g. Church/mosque or shop next to the household visited) |  |
| Interviewers Name: |  |
| Supervisor: |  |
| Edited by: |  |
| Interview Date: |  |
| Time Interview started: |  |
| Time Interview ended: |  |

**Background Information on Respondent**

| 101 | In what month and year were you born?  **Ulizaliwa mwaka upi ?** (probe for the actual month) | | Month [__ _ \| __ _] Year [_ __ \| _ __]  Don’t know month ……………………………….  Don’t know year ………………………………….. | | | | 98  98 |  |
| --- | --- | --- | --- | --- | --- | --- | --- | --- |
| 102 | How old are you? Age in completed years.  **Uko na miaka ngapi?** | | [___ \| ___] | | | |  |  |
| 103 | Have you ever attended school?  **Umewahi enda shule?** | | Yes..........................................................  No ......................................................... | | | | 1  2 |  |
| 104 | What is the highest class you completed?  **Umefika kiwango gani cha juu cha masomo?** | | Not attended school..............................  Primary . ................................................  Secondary...............................................  College/University.................................. | | | | 1  2  3  4 |  |
| 105 | 1. Do you have a mobile Phone?   **Je uko na simu?** | | Yes ..........................................................  No .......................................................... | | | | 1  2 |  |
|  | 1. If **No** in (Q105a) above, whose phone do you use?   **Kama la, ni simu ya nani unatumia?** | | My Husbands..........................................  My Relative............................................  My neighbour..................................... ..  I don’t use any phone………………… | | | | 1  2  3  4 |  |
| 106 | Do you know the CHV who serves in your area?  **Je, Unajua mhudumu wa afya katika hiki kijiji ambaye anawahudumia?**  ***Prompt: “Do you know the woman who gives out vitamin A to children under five in your area ”*** | | Yes.......................................................  No............................................**(skip to Q109)**  **If Yes, write the name of the CHV:**  ________________________________ | | | | 1  2 |  |
|  |  |  | | **Yes 1** | **No 2** | **Don’t Know 98** | | |
| 107 | Please tell me which of the following kinds of help or services does your CHV provide?  **Tafadhali unieleze baadhi ya huduma ambazo huwa mnapokea kutoka kwa hawa wahuduma wa afya kijijini**  (READ ALL RESPONSES) | **Service** | |  |  |  | | |
|  |  | 1. Health information including mothers groups? | | 1 | 2 | 98 | | |
|  |  | 1. Provide advice to pregnant women? | | 1 | 2 | 98 | | |
|  |  | 1. Provide advice to post-partum mother? | | 1 | 2 | 98 | | |
|  |  | 1. Provide advice regarding newborn? | | 1 | 2 | 98 | | |
|  |  | 1. Provide advice and treatment regarding children’s diarrhea? | | 1 | 2 | 98 | | |
|  |  | 1. Provide advice and treatment regarding children’s respiratory infection (including pneumonia)? | | 1 | 2 | 98 | | |
|  |  | 1. Supply condoms and pills? | | 1 | 2 | 98 | | |
|  |  | 1. Vitamin A for mother / child? | | 1 | 2 | 98 | | |
|  |  | 1. Provide HIV/AIDS/STI information? | | 1 | 2 | 98 | | |
|  |  | 1. Other (specify) | | 1 | 2 | 98 | | |
|  |  |  | | **Yes 1** | **No 2** | **Don’t Know 98** | | |
| 108 | Have you received advice from your CHV on any of the following topics?  **(READ ALL RESPONSES)**  **Je, umepata mafunzo yoyote kutoka kwa hawa wahudumu kwa mafunzo yafuatayo?** | 1. Seek ANC from health worker? | | 1 | 2 | 98 | | |
|  |  | 1. Take rest and avoid heavy work? | | 1 | 2 | 98 | | |
|  |  | 1. Proper, balanced diet? | | 1 | 2 | 98 | | |
|  |  | 1. Avoid alcohol and smoking during pregnancy? | | 1 | 2 | 98 | | |
|  |  | 1. Tetanus toxoid vaccination? | | 1 | 2 | 98 | | |
|  |  | 1. Obtain iron tablets? | | 1 | 2 | 98 | | |
|  |  | 1. Obtain deworming tablets | | 1 | 2 | 98 | | |
|  |  | 1. Obtain Malaria tabs | | 1 | 2 | 98 | | |
|  |  | 1. Financial preparation for your delivery? | | 1 | 2 | 98 | | |
|  |  | 1. Identifying emergency transport options? | | 1 | 2 | 98 | | |
|  |  | 1. Danger signs for pregnant woman? | | 1 | 2 | 98 | | |
|  |  | 1. Use Clean Home Delivery Kit? | | 1 | 2 | 98 | | |
|  |  | 1. Danger signs during delivery? | | 1 | 2 | 98 | | |
|  |  | 1. Make post-natal visit to a health facility or outreach clinic? | | 1 | 2 | 98 | | |
|  |  | 1. Danger signs for postpartum woman? | | 1 | 2 | 98 | | |
|  |  | 1. Use family planning following delivery? | | 1 | 2 | 98 | | |
|  |  | 1. Anyone touching newborn to wash hands with soap and water first | | 1 | 2 | 98 | | |
|  |  | 1. Wrap the newborn in a clean and dry cloth? | | 1 | 2 | 98 | | |
|  |  | 1. Do not bathe the newborn within 24 hrs? | | 1 | 2 | 98 | | |
|  |  | 1. Keep the newborn’s cord dry and clean (do not apply anything)? | | 1 | 2 | 98 | | |
|  |  | 1. Breastfeed the newborn within 1 hr. after birth? | | 1 | 2 | 98 | | |
|  |  | 1. Continue exclusive breastfeeding? | | 1 | 2 | 98 | | |
|  |  | 1. Danger signs in newborn? | | 1 | 2 | 98 | | |
|  |  | 1. Skin to skin contact? | | 1 | 2 | 98 | | |
|  |  | 1. Personal hygiene of mother? | | 1 | 2 | 98 | | |

**Interviewer: “Now, I would like to ask you some questions about when you were pregnant prior to your most recent delivery.”**

| 109 | Did you see anyone for antenatal care for the pregnancy prior to your most recent delivery?  **Je, ulipata kuona yeyote kwa huduma ya kliniki inayopeanwa kwa akina mama kabla ya kujifungua?** | | Yes.............................................................  No.............................................................. | | | | 1  2 |
| --- | --- | --- | --- | --- | --- | --- | --- |
| 110 | Whom did you see?  **Nani ambaye alikuhudumia?**  Ask: “Anybody else?” Continue until no further answers.  **(CIRCLE ALL RESPONSES GIVEN)** | | Doctor..........................................  Nurse............................................  CM .................................................  CHV .................................................  TBA .................................................  Other (Specify) .......................... | | | | 1  2  3  4  5  6 |
| 111 | How many times did you receive antenatal care from a health worker during the pregnancy prior to your most recent delivery?  **Ni mara ngapi ulipata huduma ambayo inayopeanwa kwa akina mama wajawazito ya kabla ya kujifungua kutoka kwa wahudumu wa afya vijijini**? | | Number of times ____ ____  Don’t know ............................ | | | | 98 |
| 112 | How many months pregnant were you when you first received antenatal care from a health worker for the pregnancy prior to your most recent delivery**?**  **Ulikua kwa mwezi wa ngapi wa ujauzito wako wakati ambapo ulipata huduma ya kwanza inayopeanwa kwa akina mama wajawazito kutoka kwa wahudumu wa afya?** | | Number of months ____  Don’t know.................................. | | | | 98 |
| 113  (a) | Who are the people who accompanied you at least one time to your antenatal care?  **Ni watu gani ambao ualiandamana nao ukienda kwa kliniki ya akina mama wajawazito angalau mara moja.**  Ask: “Anybody else?” Continue until no further answers.  (CIRCLE ALL RESPONSES GIVEN) | | Husband……...…………........……  Mother-in-law . . . ………....…....  Your Mother ...….................…  Other family member……  Friend / Neighbor …………….  Other (specify)…………………  Nobody (went alone)……….  Don’t know.................................... | | | | 1  2  3  4  5  6  97  98 |
| 113  (b) | Where did you receive antenatal care for your most recent antenatal care visit?  **Ni wapi ulipata huduma ya kliniki ya akina mama waja wazito kabla ya kujifungua hivi karibuni?**  **If source is hospital, health center, or clinic, write the name of the place.** | | **Insert name of institution:**  __________________________________ | | | |  |
|  |  |  | Specify if the place named above is:  Hospital.............................................  Health centre..................................  Dispensary.......................................  Clinic...................................................  TBA....................................................  Other (specify) ………………  Don’t know……………………… | | | | 1  2  3  4  5  6  98 |
| 114 | As part of your antenatal care during the pregnancy prior to your most recent delivery, were any of the following done at least once?  **Ulipokuwa ukihudhuria huduma ya kliniki ya akina mama kabla ya kujifungua, ulipata hizi huduma zifuatazo?** (Read each service, circle appropriate response) |  | | Yes  1 | No 2 | Don’t Know 98 | |
|  |  | Was your abdomen examined? | | 1 | 2 | 98 | |
|  |  | Did you receive iron tablets? | | 1 | 2 | 98 | |
|  |  | Did you receive deworming tablets? | | 1 | 2 | 98 | |
|  |  | Did you receive TT vaccination? | | 1 | 2 | 98 | |
|  |  | Was your weight measured? | | 1 | 2 | 98 | |
|  |  | Was your blood pressure measured? | | 1 | 2 | 98 | |
|  |  | Did you give a blood sample? | | 1 | 2 | 98 | |
| 115 | During any of your antenatal care visits with health workers during your pregnancy prior to your most recent delivery, were you counseled on:  **Wakati ulipokua ukihudhuria huduma ya kliniki ya akina mama wajawazito kabla ya kujifungua, je ulipata mawaidha yeyote?**  **(READ ALL RESPONSES)** | 1. Financial preparation for your delivery? | | 1 | 2 | 98 | |
|  |  | 1. Identifying emergency transport options? | | 1 | 2 | 98 | |
|  |  | 1. Arranging for blood in case of emergency? | | 1 | 2 | 98 | |
|  |  | 1. Tetanus toxoid vaccination? | | 1 | 2 | 98 | |
|  |  | 1. Danger signs during pregnancy? | | 1 | 2 | 98 | |
|  |  | 1. Delivery in a suitable health facility? | | 1 | 2 | 98 | |
|  |  | 1. All who touch the newborn need to wash hands with soap and water first? | | 1 | 2 | 98 | |
|  |  | 1. Immediate drying and wrapping of the newborn? | | 1 | 2 | 98 | |
|  |  | 1. Breastfeeding immediately after birth? | | 1 | 2 | 98 | |
|  |  | 1. Care of the newborn, particularly cleanliness, avoiding chilling, and immediate breastfeeding? | | 1 | 2 | 98 | |
|  |  | 1. Family planning? | | 1 | 2 | 98 | |

| 116 | Did you discuss planning for your delivery with your husband while you were pregnant?  **Je, Wakati ulikuwa mjamzito ulipata kupanga kuhusu kujifungua kwako na mume wako?** | Yes ......................................................  No ........................................................ | 1  2 |
| --- | --- | --- | --- |
| 117 | Were you given or did you buy any iron/folic acid tablets when you were pregnant prior to your most recent delivery?  **Ulipewa dawa / tembe ya kuongeza madini au damu mwilini ama uliweza kununua wakati ulikuwa mja mzito?** | Yes ..................................................  No ....................................................  Don’t know …………………… | 1  2  8 |
|  | During your pregnancy prior to your most recent delivery, did you receive deworming tablets?  **Je, ulipata dawa za minyoo wakati ulikuwa mja mzito?** | Yes......................................................  No....................................................... Don’t know ……………………. | 1  2  98 |
|  | During your pregnancy prior to your most recent delivery, did you eat less than usual, about the same amount as usual, or more than usual?  **Je wakati wa ujauzito wako, ulikula chakula kiwango cha chini, kawaida ama kiwango cha juu kushida hapo awali?** | Less than usual .............................  Same as usual ...............................  More than as usual ....................  Don’t know ................................... | 1  2  3  98 |
|  | Please tell me where you should go for health services if you have danger signs while you are pregnant? **(CIRCLE ALL RESPONSES GIVEN)**  **Tafadhli nieleze ni wapi unaweza enda kupata huduma kama umepata dalili za hatari wakati uko mja mzito** | Hospital ........................................  Health Centre..............................  Dispensary...................................  Private Clinic ..............................  Other (specify) ……………  Don’t know…………………… | 1  2  3  4  5  98 |
|  | What are the symptoms during pregnancy indicating the need to seek immediate care?  **Ni dalili zipi wakati uko mja mzito ambazo** **zinaonyesha umuhimu wa kupata huduma kwa haraka?**  **Ask: “Any others?” Continue until no further answers. (CIRCLE ALL RESPONSES GIVEN)** | Vaginal bleeding (any amount) …..  Severe lower abdominal pain.......  Severe headache..................................  Convulsions........................................... Blurred vision and swelling of hands and face......................................  Other (specify) __________________  Don’t know......................................... | 1  2  3  4  5  6  98 |
|  |  |  |  |

| 122 | During your pregnancy prior to your most recent delivery, did you experience any of the following problems at any time?  **Je, wakati ulikua mja mzito, ulipata shida yeyote wakati wowote?**  **(Read out all responses one after another) (Record all responses accordingly.)** | 1. Blurred vision? | Yes (1) | No (2) |
| --- | --- | --- | --- | --- |
|  |  | 1. Severe lower abdominal pain? | 1 | 2 |
|  |  | 1. Severe headache? | 1 | 2 |
|  |  | 1. Convulsions? | 1 | 2 |
|  |  | 1. Swelling of the hands, body or face | 1 | 2 |
|  |  | 1. Any vaginal spotting or bleeding? | 1 | 2 |
|  |  | 1. None of the above (skip to 124) | 1 | 2 |
|  |  | 1. **Other (specify)………………** | 1 | 2 |
| 123 | During your pregnancy prior to your most recent delivery, what did you do or whom did you consult for the problems that you stated above?  **Wakati ulikua mja mzito,ulifanya nini ama ulimuuliza mtu yeyote kwa shida ambazo umetaja?**  **Prompt: “anything else?”**  **(CIRCLE ALL RESPONSES GIVEN)** | Traditional treatment at home.…........…….........  Given medicine at home …………………………….  Hospital …………………………………………………....  Health Centre ……………..….....................................  Private Hospital/Clinic/N. Home…………………  Bought medicine from pharmacy …...................  Consulted CHV …………………....................................  Consulted Community Midwife .… …………  Consulted a TBA ………………………………………..  Consulted other HW ………………………………  Consulted relative/neighbor/friend ………  Other (specify)………………………………………  Nothing ……………………………................................ | | 1  2  3  4  5  6  7  8  9  10  11  12  97 |

**Interviewer: “Now, I would like to ask you some questions about your most recent delivery.”**

|  | Where did you plan to give birth in your last pregnancy?  **Je ulipanga kujifungulia wapi?** | I did not plan anything…………………..  I planned to deliver at Hospital ………  At Community midwife Clinic …………  At TBA clinic ………………………………….  At home …………..…………………………  Other (specify) …………………………… | 1  2  3  4  5  6 |
| --- | --- | --- | --- |
|  | Where did you give birth in your most recent delivery?  If source is hospital, health center, or clinic, write the name of the place.  **Je, ulijifungulia wapi hivi karibuni?**  Probe to identify the type of source and circle the appropriate code to the right. _________________________________ **NAME OF PLACE** | Hospital .......................................................  Health Centre.............................................  Private Clinic/N.Home ................................  At Community midwife Clinic……………  TBA ............................................................  Your home …………………………..………  Other home ………………………….………  Other (specify) ……………………………… | 1  2  3  4  5  6  7  8 |
|  | Who assisted with your most recent delivery? Prompt: “Anybody else?”  **Je, kuna mtu yeyote ambaye alikusaidia wakati ulikua unajifungua huyu mtoto wako wa mwisho?**  **(CIRCLE ALL RESPONSES GIVEN)** | Doctor…………………………….………………  Nurse in health facility …….……………....  Clinical officer ………………………………….  Community Midwife ………………………..  CHW ……………………………………………  TBA……………………..............….....................  Relative/Friend……………………..……….  No body/Myself……………………..………  Other (Specify) ……………………..……… | 1  2  3  4  5  6  7  8  9 |
|  | Who else was present at the delivery outside the room where the delivery took place? Prompt: “Anybody else?”  **Ni nani mwingine alikuwepo nje ya jumba ambapo ulikua unajifungulia?**  **(CIRCLE ALL RESPONSES GIVEN)** | TBA……………………….................................... CHV……………………..……………………….. Friends/Neighbors......……………………..  Mother-in-law......…………………………..….  Husband....………….……………………………  Mother....…………………………………………  Father......……………...…………………………  Other relative ……………………………….…  Other (specify)…………………………………  Nobody ………………………….........................  Don’t know/cannot remember.................. | 1  2  3  4  5  6  7  8  9  97  98 |
|  | During your pregnancy prior to your most recent delivery, was your child delivered by caesarean section? Prompt: Ask “did a doctor cut open your abdomen to deliver the baby?”  **Je huyu mtoto ambaye umejifungua hivi karibuni, alizaliwa kwa njia ya upasuaji? Au daktari alikukata ili mtoto azaliwe?** | Yes……………………………........…………………  No……………………………........……………….. | 1  2 |
|  | 1. During your pregnancy prior to your most recent delivery, comment of the nature of delivery. Was it a normal delivery or you experienced complications?   **Je, wakati ulipokuwa unajifungua, ulijifungua kwa kawaida ama ulipa tatizo lolote?**   1. If you experienced complications, what were they?   **Kama ulipata matatizo, yalikuwa gani?** | Normal (spontaneous vaginal delivery) …  Experienced complications ………………… | 1  2 |
|  |  | Arm prolapse………………………………………..  Shoulder presentation …………………………  Other parts of body (mention………….…….)  Don’t know ………………………….. … | 1  2  3  98 |
|  | If you delivered in Hospital, Was baby reviewed before you were discharged from the hospital  **Kama ulijifungulia hospitalini, je mtoto alikaguliwa kabla hamjaruhusiwa kwenda nyumbani?** | Yes……….……(**Skip to Q132)**  No……………………………........….. | 1  2 |
|  | FOR BIRTHS IN OTHER HEALTH FACILITY (besides hospital), ASK: Before you were discharged, did any health care provider check on your baby?  **Je, kuna mhudumu wa afya alikagua mtoto kabla hamruhusiwa kwenda nyumbani?** | Yes………………………………….  No.……………….………………… | 1  2 |
|  | How long after delivery did the first check-up take place?  **Ni muda gani baada ya kujifungua ambapo uliangaliwa kwa mara ya kwanza?** | MINUTES (insert No. of MINUTES) ……  HOURS (insert No. of HRs) ........………  DAYS (insert No. of days) ..........................  Don’t know….…………………………… | 98 |
|  | Who checked on your health at that (FIRST) time? PROBE FOR MOST QUALIFIED PERSON. **(countercheck Q127 again)**  **Ni nani aliweza kukagua au kupima afya yako kwa mara ya kwanza?** | Doctor.......................................................  Nurse in a health facility....................  Clinical Officer.......................................... Community midwife............................  CHW .........................................................  TBA ……………………….…………………  Other (specify) ………………………  Don’t know / don’t remember …… | 1  2  3  4  5  6  7  98 |
|  | How long after delivery did the second  Check-up of your health take place?  **Ni muda gani baada ya kujifungua ambapo ulikaguliwa au kupimwa tena kwa mara a pili?** | MINUTES (insert No. of MINUTES) …  HOURS (insert No. of HRs) ........……  DAYS (insert No. of days) .......................  **Weeks (insert No. of wks) .....**  **Months (insert No. of mth) ........**  Don’t know….……………… | 98 |
|  | Who checked on your health at that (SECOND) time? PROBE FOR MOST QUALIFIED PERSON.    **Ni nani alipima au kugagua afya yako mara ya pili?** | Doctor .......................................................  Nurse ........................................................  Clinical Officer ........................................... Community Midwife .................................  CHW ......................................................... TBA……………………….………  Other (specify) _____________________  Don’t know / don’t remember ……….... | 1  2  3  4  5  6  7  98 |
|  | Where did these checks take place? Prompt: “Anywhere else?” **(CIRCLE ALL RESPONSES GIVEN)**  **Ulipimwa au kukaguliwa ukiwa wapi?** | Hospital………..……………………….  Health Centre…………………...….......... Dispensary…………………..….………….  Private Hospital/clinic/N. Home….  NGO clinic……………….…………………  Home …………………………………………  Other (specify)_____________________ | 1  2  3  4  5  6  7 |

**Interviewer: “Now, I would like to ask you some specific questions pertaining to the baby immediately following the delivery.”**

|  | What instrument was used to cut the cord?  **Ni kifaa kipi ambacho kilitumika kukata kitovu?** | New Blade..……………………..………………..  Boiled Blade ……………………………………..  Unboiled used blade………………………… Knife………………………………………………….  Scissor…………………………….………………….  Other (specify) __________________  Don’t know (**Skip to Q139)**…………… | 1  2  3  4  5  6  98 |
| --- | --- | --- | --- |
|  | Was the instrument used to cut cord boiled prior to use?  **Je kile kifaa ambacho kilitumika kukata kitovu kilichemswa kabla ya kutumiwa?** | Yes ..........................................................  No...........................................................  Don’t Know/Can’t Remember............…… | 1  2  98 |
|  | What was used to tie the cord?  **Ni nini ilitumika kufunga kitovu?** | New ties ………………………………………………  Boiled string or thread ………………..........  Unboiled used string or thread ……..……  Other (specify) …………………………………….  4 Don’t know………………..............….…….. | 1  2  3  4  98 |
|  | On what surface was the cord cut on?  **Ni mahali gani ambapo kitovu ilikatiwa?** | Plastic disc..………..…………...………………….  Metal coin.. ………..……………….…............ Wood……………..….……….………................  Other (specify)………………………………….... Nothing...…………………………………………….  Don’t know.……………………….................. | 1  2  3  4  97  98 |
|  | Did the person who handled the baby, assisting with the delivery, wash hands with soap and water first?  **Je mtu ambaye alishika mtoto, kukusaidia kujifungua aliosha mikono yake kwa maji na sabuni kwanza?** | Yes ………………………………....…………………  No …………………………..……....……………….  Don’t know ……………………………………….. | 1  2  98 |
|  | Did anybody apply anything on the stump after the baby’s cord was cut?  **Je kuna mtu alipaka chochote kwenye kitovu ya mtoto baada ya** **kitovu kukatwa?** | Yes ………………………………....………………..  No …………………………..……....… skip to 144  Don’t know …………………………skip to 144 | 1  2  98 |
|  | What did they apply ?  **Je, walipaka nini?**  (Prompt: “Anything else?”)  (CIRCLE ALL RESPONSES GIVEN) | Oil……………………………….…………………….. Ash…………………………….………………………. Ointment/powder……………..……………….  Animal dung…………………...………………… Turmeric/turmeric powder………...………  Other (specify) ___________________  Don’t know…………………………………………. | 1  2  3  4  5  6  98 |
|  | If a special medicine were available for preventing infections of the cord stump, do you think you would want to use it?  **Je, kama kuna dawa spesheli ya kuzuia magonjwa ya kitovu ya mtoto unafikiria ungependa kuitumia?** | Yes ………………………………..……………………  No ……………………………………………………..  Don’t know..........…………………………….. | 1  2  98 |
|  | Was your baby wiped off/dried before the placenta was delivered?  **Je mtoto wako alipanguzwa kabla ya kondo la nyuma au placenta kutolewa?** | Yes ………………………………..………………….  No …………………………………………………….  Don't know…......………………………………. | 1  2  98 |
|  | Was your baby wrapped in cloth or put on mother's body and covered with cloth before the placenta was delivered?  **Je,mtoto wako alifungiwa kwenye nguo ama kitambaa ama kuwekwa karibu nawe kabla ya kondo la nyuma au placenta kutolewa?** | Yes …………………………………………………… No………………………………………………………  Don't know…......…………………..…………… | 1  2  98 |
|  | What was the condition of the cloth, which was used for wrapping the baby?  **Nguo au kitambaa iliyotumika kumfungia mtoto ilikua kwa hali gani?**  (Probe: “Anything else?”)  (CIRCLE ALL RESPONSES GIVEN) | Clean cloth. ……………………………………….  Dry cloth. ……………………………...............  New cloth…. …………………….……………….  Used cloth…. …………………………………….  Wet cloth……………………………………………  Other (specify)……………………................  Don’t know ...…………………………………….. | 1  2  3  4  5  6  98 |
|  | Where was the baby placed before the placenta was delivered?  **Mtoto aliwekwa wapi kabla ya kondo la nyuma au placenta kutolewa?** | On the floor ………………………………………..  On the cot ……………………………..............  Beside or on the mother's body…………  With someone else …………………………..  Other (specify) ___________________  Don’t know ……………………………………….. | 1  2  3  4  5  98 |
|  | How long after birth was your baby bathed for the first time?  Ni muda gani baada ya kujifungua ambapo mtoto aliogeshwa kwa mara ya kwanza? | Within 1 hour …………………………………….  2-24 hours …………………………………………  After 24 hours ………………………………….. Don't know......................................….. | 1  2  3  98 |
|  | Did you ever breastfeed  (NAME)?  **Je,Ulimnyonyesha mtoto?** | Yes……………………………………………………… No…………………………………………skip to 152 | 1  2 |
|  | If Yes, how long after birth did you first put (NAME) to the breast?  **Kama ndio ulimpatia mtoto maziwa ya matiti baada ya muda gani?** | During the first hour after delivery.……..  More than 1 hour. ..……………….……………  Don’t know………………………..……………….. | 1  2  98 |
|  | Did you give (NAME) the first milk that came from your breasts?  **Je, Uliweza kumpatia mtoto maziwa ya matiti?** | Yes……………………………………………………… No…………………………………………………….. | 1  2 |
|  | In the first three days after birth was (NAME) given anything to drink other than breast milk?  **Kwa siku tatu baada ya kuzaliwa, mtoto alipewa chochote kunywa isipokuwa ya maziwa ya mama?** | Yes ……………………......……………………….. No……………………......……...…................ | 1  2 |

**Interviewer: “Now, I would like to ask you some questions about the health of your child during the month after your most recent delivery.”**

|  | What are the symptoms of the infant within one month after delivery indicating the need to seek immediate health care?  **Ni dalili gani ambazo zinaonyesha kwamba mtoto mchanga wa mwezi mmoja baada ya kuzaliwa anahitaji huduma ya dharura au haraka ya kiafya?**  (Prompt: “Any other symptoms?”)    (CIRCLE ALL RESPONSES GIVEN) | Poor sucking or not able to ………….……………………………..  Fast breathing ..................……. ……………………………………  Severe chest in-drawing............................................... Hypothermia………………................……………………………… Fever.............................................................................  Difficult to wake/lethargic/unconscious.......................  Pustules on skin 1 large or more than 10 small ones…..  Severe umbilical infection redness of skin the cord/ foul smelling discharge OR bleeding from the cord………….....  Other (specify)………………………………………………………………  Don’t know………………………….......................................... | 1  2  3  4  5  6  7  8  9  98 |
| --- | --- | --- | --- |
|  | Probe about the outcome of pregnancy  Interviewer: circle appropriate code | Baby still alive………………………………………………………..  Baby born alive, then died at 2+ months…..………………  Baby born alive, then died at 0-1 months….………………  Baby still born….…………………………………………………… | 1  2  3  4 |

**For mothers/care givers who are pregnant at the time of interview: Birth Preparedness**

**Interviewer: “Now, I would like to ask you some questions about how you are prepared for the arrival of your next child.”**

|  | Have you made any preparations for your delivery?  **Umefanya matayarisho yoyote ya kujifungua?** | Yes …………………………..………………………….  No ……………………………………….skip to 158 | 1  2 |
| --- | --- | --- | --- |
|  | If Yes, what preparations have you made for the delivery? (Prompt: “Did you do anything else to prepare?”)  **Kama ndio, ni matayarisho gani umefanya?**  (CIRCLE ALL RESPONSES GIVEN) | Financial ……………………………..................  Transport …………………………………………….  Food …………………………………...................  Identification of skilled birth attendant...  Identification of facility ….…….…...………..  Blood …………….……………………………………..  Clean delivery kit...……….........................  Clothes for new born.............................. Other (specify) ___________________ | 1  2  3  4  5  6  7  8  9 |
|  | Have you arranged for a place to deliver your child?  **Je,umepanga mahali ambapo utajifungulia mtoto?** | Yes …………………………..…...…………………….  No ……………………………………………………….. | 1  2 |
|  | Where have you planned to deliver your child?  **Umepanga kujifungulia wapi?** | Hospital................................................. Health center .............................………….  Dispensary............................................. Private Hospital/clinic /N. home............ NGO clinic ……………………………………………  At home ________________________  Other (specify) ___________________ | 1  2  3  4  5  6  7 |
|  | Do you plan to make preparation for care of the mother and newborn during the first month following the delivery?  **Je,Unapanga kufanya matayarisho yoyote kwa huduma yako mwenyewe pamoja na ya mtoto wako mchanga katika mwezi wa kwanza baada ya kujifungua?** | Yes …………………………..…...…………………….  No …………………………………………(skip to 162) | 1  2 |
|  | If Yes, what preparations will you make for care of the mother and newborn during the first month following the delivery?  **Kama ndio, ni matayarisho gani utafanya kwako wewe mwenyewe pamoja na ya mtoto mchanga kwa mwezi wa kwanza baada ya kijifungua?**  (Prompt: “Have you done anything else to prepare?”) (CIRCLE ALL RESPONSES GIVEN) | Financial ……………………………..................  Identification of health worker …...…..... Identification of facility ….…….…...………..  Blood …………….…………………………………….  Other (specify) ___________________ | 1  2  3  4  5 |
|  | Did you make any preparation for emergencies during pregnancy, delivery, or after delivery?  **Ulifanya matayarisho yoyote ya dharura wakati ulikuwa mja mzito, wakati wa kujifungua ama baada ya kujifungua?** | Yes …………………………..…...…………………….  No …………………………………………(skip to 164) | 1  2 |
|  | If Yes, what kind of preparations have you made? (Prompt: “Anything else ?”)  **Kama ndio, ni matayarisho gani umefanya?**  (CIRCLE ALL RESPONSES GIVEN) | Financial ……………………………..................  Transport ……………………………………………. Identification of health worker …..…....... Identification of facility ….…….…...………..  Blood …………….…………………………………….  Other (specify) ___________________ | 1  2  3  4  5  6 |

**New born care practices**

|  | **Cord care:** Pease describe how mothers in this community take care of the cord for quick recovery.  What substances are placed at the stump of the cord to assist healing?  **Tafadhali nielezee jinsi akina mama wa kijiji hiki hufanya kulinda kitovu ili imepone haraka, ni nini haswa hao huweka kwenye kitovu kusaidia ili ipone?** | Nothing……..……………...........…………………  Cow dung……………..........………………….  Mud……..……………...........…………………  Traditional leaves…..........………………….  Medicine……..……………...........…………………  Other (Specify)…..........…………………. | 1  2  3  4  5  6 |
| --- | --- | --- | --- |
|  | **Rashes:** When a new born baby develops rashes, what do mothers in this community do to clear the rash?  **Wakati mtoto mchanga anapopata kujikunakuna ama upele, akina mama wa kijiji hiki hufanya nini ili kumaliza kujikunakuna?** | Wash the baby with water……………………  Wash the baby with leaves……………………  Apply oil .......…………….  Take baby to hospital….......………………  Other (specify)…...........………………………. | 1  2  3  4  97 |
|  | **Washing baby:** When do mothers in this community wash their babies for the first time after delivery?  Prompt: “anywhere else?” until no more responses.  **Ni wakati gani akina mama wa kijiji hiki huosha watoto wao wachanga kwa mara ya kwanza baada ya kujifungua?** | Immediately after birth…………………  After one day …………………………………  After 2 days ………………………………….  After one week …………..  Others (specify) …………………………… | 1  2  3  4  5 |
|  | With what do mothers use to wash their babies?  **Akina mama hutumia nini kuosha watoto wao wachanga?** | _____________________________ |  |
|  | What do mothers in this community give to their new born babies for food?  Prompt: “anywhere else?” until no more responses.  **Akina mama katika kijiji hiki huwapatia nini watoto wao wachanga kama chakula?** | Breast milk……………..….………………….. Water………………………..……………..  Cow milk………………………..……………..  Porridge………………………..……………..  Others (specify)……………………………. | 1  2  3  4  5 |
|  | Do mothers in this community make or buy baby clothes prior to delivery?  **Akina mama katika kijiji hiki huwa wananunua nguo za mtoto kabla ya kujifungua?** | Yes……..………………..….………………….. No………………………………..…………….. |  |
|  | How do mothers in this community cover their babies soon after delivery?  **Akina mama katika kijiji hiki huwafunika vipi watoto wao wachanga baada ya kujifungua?** | Baby clothes bought…………………………  Lesso……….…………….........  Old clothe ------------------------------------  Clothe provided by midwife………....……  Other (Specify) ……………..……………… | 1  2  3  4  5 |
|  | Please list three common cultural practices that mothers do to protect the new born baby?  **Tafadhali nieleze mambo matatu ya kitamaduni ambayo kina mama hufanya kuwalinda watoto wao wachanga? Probe** | 1._____________________________  2._____________________________  3._____________________________ |  |

**Information and Communication Sources**

|  | In the past three months, have you seen, heard, or read anything about the importance of delivering in a suitable health facility?  **Kwa miezi tatu iliyopita, umewahi kuona, kusikia ama kusoma mahali kuhusu umuhimu wa kujifungua katika hospitali au kituo cha afya?** | Yes……..……………...........………………… No……………………..........………(skip to 174) | 1  2 |
| --- | --- | --- | --- |
|  | Through which media did you see, hear or read about this information?  **Ni wapi ambapo ulipata ujumbe huu?** | TV…………...........……………………………  Radio…………...........………………………  Newspaper/magazine.......…………….  Mobile Phone………….......………………  CHV…………...........………………………….  CM…………...........……………………………  Nurse…………...........………………………..  Doctor…………...........………………………  Other (specify) ..……………………….  I have not heard anything……......…… | 1  2  3  4  5  6  7  8  9  97 |
|  | Please tell me where you saw or heard a message on importance of delivering in a suitable health facility. Prompt: “anywhere else?” until no more responses.  **Tafadhali niambie pahali uliona ama kusikia ujumbe wa umuhimu wa kujifungua katika hospitali au kituo cha afya?** | CHV flip chart ………………………………  From CHV …………………………………  From TBA ………………………………….  From health facility worker…………..  Posters, pamphlets, leaflets …………  Newspaper ……………………………………  Radio …………………………………………….  TV …………………………………………………  Others (specify) …………………………… | 1  2  3  4  5  6  7  8  9 |
|  | Have any friends, family members or other acquaintances spoken with you informally during the past two months about importance of delivering in a suitable health facility?  **Kuna marafiki, watu wa familia ama mtu yeyote ambaye amepata kuongea na wewe kuhusu umuhimu wa kujifungulia katika hospitali au kituo cha afya kwa miezi miwili iliyopita?** | Yes……..………………..….………………….. No………………………………..…………….. | 1  2 |
|  | Who should advice mothers on how to take care of their new born babies?  **Ni nani haswa anafaa kuwapatia akina mama mawaidha kuhusu jinsi ya kuwatunza watoto wachanga?** | Doctor…………………………….…………………………  Nurse in health facility……….…………….........  Clinical officer ------------------------------------  Community Midwife ………………………....……  CHV …………………………………………………………  TBA……………………..............….....................  Relative/Friend……………………..…………………  Myself……………………..………………………………  No body…………………………………………………  Other (Specify) ……………………..……………… | 1  2  3  4  5  6  7  8  9  10 |
