# Supplementary material for: Evaluating quality neonatal care, call Centre service, tele-health and community engagement in reducing newborn morbidity and mortality in Bungoma county, Kenya
Source: BMC Health Serv Res. 2018 Jun 25;18:493. doi: 10.1186/s12913-018-3293-5 (PMC6019716; doi:10.1186/s12913-018-3293-5)
Supplement: Supplementary file 2 — Tool for Service Provider - Client Observation. (DOCX 103 kb) [file 12913_2018_3293_MOESM2_ESM.docx]

| **SERIAL NUMBER** | ___\| ___\| ___\| |
| --- | --- |

**COLLABORATIVE NEW BORN SUPPORT PROJECT**

**Tool for Service Provider - Client Observation**

1. Facility Name ______________________________ Code_________(Insert Code from the list below)

| **Health Facility** | **Code** | **Health Facility** | **Code** |
| --- | --- | --- | --- |
| Webuye county Hospital |  | Kapsakwony sub-county hospital | 06 |
| Sirisia Sub- County Hospital |  | Bumula sub-county hospital | 07 |
| Kimilili Sub County hospital |  | Chwele sub County Hospitals. | 08 |
| Naitiri Sub county Hospital |  | Sinoko Sub-County Hospital | 09 |
| Bungoma County Referral hospital |  |  |  |

1. Sub-County name for selected facility …………………………………… Code_________(Insert Code from list below)

| **Name of Sub-County** | **Code** | **Name of Sub-County** | **Code** |
| --- | --- | --- | --- |
| Bumula |  | Tongaren | (6) |
| Kanduyi |  | Mt. Elgon | (7) |
| Kabuchai |  | Webuye East | (8) |
| Sirisia |  | Webuye West | (9) |
| Kimilili |  |  |  |

## Status of the Hospital at the time of data collection: Circle appropriate code: Phase I=01; Phase II =02

## Observer to establish the designation of the provider giving services to clients and to circle appropriate code

- 1. Enrolled Nurse/Midwife (Certificate)……………………. ………………………1
  2. Registered Nurse (Diploma)…………………………………….…………..2
  3. Degree Nurses (Degree e.g. BScN) .……………………….…………..3
  4. Clinical Officers (Diploma)………………………………………………….4
  5. Medical Officers (Degree)…………………………………………………….5
  6. Paediatrician (Degree)………………………………………...………….6
  7. Others (specify) ..……………………………………………………………………..……………….7

1. Section/Unit where client-provider interaction is being observed (Circle appropriate box)*.*

| Nursery | 01 | Maternity/Delivery Room | 02 | MCH | 03 | Post Natal ward | 04 | Paediatrics ward | 05 | OPD | 06 |
| --- | --- | --- | --- | --- | --- | --- | --- | --- | --- | --- | --- |

1. Date of Interview (DAY, MONTH, YEAR E.G. 02/03/16) [___|___/___|___/___|___]

| 1. Interview Result | Completed | 01 |
| --- | --- | --- |
|  | Partially Completed | 02 |
|  | Refused | 03 |
|  | Other (specify)_______________________ | 96 |

1. Name of Interviewer ………………………………………………………………... Signature ………………………………..
2. Supervisor’s Name ………………………………………………….…….…………..… Signature ……………………………….

**OBSERVATION TOOL FOR HEALTH PROVIDERS**

**INSTRUCTIONS TO THE OBSERVER:**

- **Obtain permission from the facility administration and consent from a provider and the mother or caretaker of the baby before observing the consultation.**
- **When observing be as discreet as possible and on no account become involved in the interaction.**
- **Make sure the provider knows that you are not there to evaluate him/ her and that you are not an “expert” who can be consulted during the observation.**
- **Try to sit in a position such that you are behind the patient but not directly in the view of the provider. Make notes as quickly as possible. For each of the items circle the answer that most appropriately reflects your assessment of what happened during the interaction.**

| ***Part A: GREETING AND ASSESSING CLIENT*** | | | | |
| --- | --- | --- | --- | --- |
| ***TIME CONSULTATION STARTED: HOUR MINUTE*** | | | | |
| ***CIRCLE THE APPROPRIATE CODE*** | | | | |
|  |  | **YES** | **NO** | **N/A** |
|  | **DOES THE PROVIDER GREET THE MOTHER/ CARETAKER IN A FRIENDLY/RESPECTFUL MANNER?** | 1 | 0 | **8** |
|  | **DOES THE PROVIDER:** | **YES** | **NO** | ***N/A*** |
|  | ENSURE PRIVACY? | 1 | 0 | 8 |
|  | ENSURE CONFIDENTIALITY? | 1 | 0 | 8 |
|  | TELL MOTHER/ CARETAKER WHAT’S GOING TO BE DONE? | 1 | 0 | 8 |
|  | ENCOURAGE HER/HIM TO ASK QUESTIONS? | 1 | 0 | 8 |
|  | **ARE THE FOLLOWING AREAS DISCUSSED/MENTIONED DURING THE CONSULTATION (OBSERVE AND CIRCLE IF MENTIONED):** | **YES** | **NO** | ***N/A*** |
|  | PURPOSE OF MOTHER/ CARETAKER VISIT TO THE HEALTH FACILITY | 1 | 0 | 8 |
|  | BABY’S AGE | 1 | 0 | 8 |
|  | NAME OF THE MOTHER/CARETAKER | 1 | 0 | 8 |
|  | BABY’S PRESENTING SYMPTOM/SIGNS | 1 | 0 | 8 |
|  | HISTORY OF BABY’S PRESENT ILLNESS | 1 | 0 | 8 |
|  | EXPLORE MEDICAL/ SURGICAL HISTORY RELATED TO BABY’S ILLNESS | 1 | 0 | 8 |
|  | UNDERSTANDING PROGRESSION OF SYMPTOMS AND SIGNS OF BABY’S ILLNESS | 1 | 0 | 8 |
|  | HISTORY OF PREGNANCY (PREGNANCY ORDER, DATE OF DELIVERY, MATURITY, DURATION OF LABOUR, TYPE OF DELIVERY, BABY’S WEIGHT) | 1 | 0 | 8 |
|  | HISTORY OF BABY’S IMMUNIZATION STARTED | 1 | 0 | 8 |
|  | ASSESS BABY’S FEEDING PRACTICE | 1 | 0 | 8 |
|  | PERFORM PHYSICAL EXAMINATION TO ASSESS BABY’S CONDITION | 1 | 0 | 8 |
|  | CHECK ON THE UMBILICAL CORD | 1 | 0 | 8 |
|  | **Did the Provider perform the following actions regarding care of the cord?** | **Skip to 14 if provider did nothing** | | |
|  | Washed hands before and after cord care | 1 | 0 | 8 |
|  | Put nothing on the stump | 1 | 0 | 8 |
|  | Folded nappy (diaper) below stump | 1 | 0 | 8 |
|  | Kept cord stump loosely covered with clean clothes | 1 | 0 | 8 |
|  | Did not bandage the stump or abdomen | 1 | 0 | 8 |
|  | Washed it with clean water and soap and dried it thoroughly with clean cloth **(If stump was soiled)** | 1 | 0 | 8 |
|  | Chlorhexidine | 1 | 0 | 8 |
|  | Did **not** touch the stump with bear hands ( i.e. Provider wore a pair of gloves) | 1 | 0 | 8 |
|  | Explained that mother should avoid touching the cord unnecessarily | 1 | 0 | 8 |
|  | Advised the mother to seek medical care if the umbilicus became red or started draining pus or blood | 1 | 0 | 8 |
|  | **Examination of the Newborn: Does the provider look at the following systems, organs or functions:** |  |  |  |
|  | Respiration *(The normal respiratory rate is 30-60 breaths per min)* | 1 | 0 | 8 |
|  | Colour *(Nb. Normal colour of the baby is pink)* | 1 | 0 | 8 |
|  | Heart rate (Normal, 100 - 160 beats per minute) | 1 | 0 | 8 |
|  | Body Temperature (Normal, 36.5 °C - 37.5 °C) | 1 | 0 | 8 |
|  | Posture and Movements (observed or history of). *(The normal resting posture of a term newborn baby comprises loosely clenched fists and flexed arms, hips, and knees.* | 1 | 0 | 8 |
|  | Muscle tone and level of alertness. (*The normal newborn baby ranges from quiet to alert and is consolable when upset. The baby is arousable when quiet or asleep).* | 1 | 0 | 8 |
|  | Limbs (position and movement of limbs, club foot, extra finger(s) or toe(s), | 1 | 0 | 8 |
|  | Skin (*baby’s skin on the trunk, abdomen, back may peel off after the first day).* | 1 | 0 | 8 |
|  | Umbilicus *(The normal umbilicus is bluish-white in colour on day 1. It then begins to dry and shrink and falls off after 7 to 10 days).* | 1 | 0 | 8 |
|  | Eyes (looks for discharge e.g. pus) | 1 | 0 | 8 |
|  | Head and Face (*The normal newborn baby’s head may be moulded from a vertex birth; this will resolve spontaneously over a period of three to four weeks)* | 1 | 0 | 8 |
|  | Mouth and Nose (provider looks for Cleft lip, Tongue tie, Cleft palate, Thrush, Central cyanosis (blue tongue and lips); nasal discharge | 1 | 0 | 8 |
|  | Abdomen and Back ( provider looks for defects e.g. abdominal distension, Spina bifida -through which the meninges and/or spinal cord may protrude) | 1 | 0 | 8 |
|  | Weight (The normal birth weight is between 2.5kg – 4.0kg) | 1 | 0 | 8 |
|  | Anus and Stool *(It is normal for a baby to have six to eight watery stools per day. A breastfed baby can stay for a number of days before passing stool and this should not cause any worry so long as the baby is happy and comfortable).* | 1 | 0 | 8 |
|  | Genitalia and urine *(Vaginal bleeding and discharge in the female newborn baby may occur for a few days during the first week of life and is not a sign of a problem)* | 1 | 0 | 8 |
|  | \| Feeding \|  \| \| --- \| --- \| | 1 | 0 | 8 |
|  | **Breastfeeding management: In the delivery room: Does the Provider:** |  |  |  |
|  | Initiate breastfeeding as soon as possible, within one hour of delivery | 1 | 0 | 8 |
|  | Give baby to mother to hold even if not breastfeeding | 1 | 0 | 8 |
|  | Advise the mother to let the baby rest on the mother’s chest in skin-to-skin contact | 1 | 0 | 8 |
|  | Tell the mother to put the baby to her breast when the baby seems to be ready | 1 | 0 | 8 |
|  | Show the mother how to hold her baby while breastfeeding | 1 | 0 | 8 |
|  | Check that the position and attachment are correct at the first feed. | 1 | 0 | 8 |
|  | Advise the mother to let the baby release the breast then offer the second breast | 1 | 0 | 8 |
|  | Advise the mother on what should happen if the baby does not feed in 1 hr. or so? | 1 | 0 | 8 |
|  | **RESUSCITATION OF THE NEWBORN DUE TO BIRTH ASPHYXIA**  **(Circle: YES if neonate has this condition; and NO if not)**  Resuscitation is meant to establish the heart and lung function following cardio-respiratory arrest. Birth Asphyxia is defined as failure to initiate and sustain breathing at birth. For many babies, the need for resuscitation cannot be anticipated before delivery. Therefore health providers need to be prepared for resuscitation at every delivery.  **During preparation for resuscitation, does the Provider check and arrange equipment/supplies at all times as follows:** |  |  |  |
|  | A firm stable surface | 1 | 0 | 8 |
|  | Source of heat e.g. heater, heater lamp or resuscitaire | 1 | 0 | 8 |
|  | Adequate lighting | 1 | 0 | 8 |
|  | Source of oxygen, flow meter, tubing and key | 1 | 0 | 8 |
|  | Suction equipment i.e. suction machine, suction catheters sizes F6, 8, 10 | 1 | 0 | 8 |
|  | Ambu bag (500mls) | 1 | 0 | 8 |
|  | Face masks sizes 0 and 1, preferably round | 1 | 0 | 8 |
|  | Wall clock | 1 | 0 | 8 |
|  | At least two pieces of warm dry linen | 1 | 0 | 8 |
|  | Syringes and needles/swabs, (preferably 1ml, 2ml and 10mls) | 1 | 0 | 8 |
|  | Stethoscope | 1 | 0 | 8 |
|  | Airways sizes: 000,00,0 | 1 | 0 | 8 |
|  | Nasal prongs | 1 | 0 | 8 |
|  | Nasogastric tube size F4, F6 and F8 (may be used as umbilical catheter) | 1 | 0 | 8 |
|  | Scissors and tape | 1 | 0 | 8 |
|  | Other equipment (e.g. Laryngoscope with extra batteries and bulb, blades, Endotracheal tube) | 1 | 0 | 8 |
|  | Other drugs e.g. Adrenaline (epinephrine) , 10% dextrose and normal saline | 1 | 0 | 8 |
|  | Penguin sucker | 1 | 0 | 8 |
|  | **Neonatal Resuscitation Procedure**  **Does the provider:** | 1 | 0 | 8 |
|  | Dry the baby, remove wet clothing and wrap baby in dry warm clothes? | 1 | 0 | 8 |
|  | Observe the baby’s breathing, colour and activity? | 1 | 0 | 8 |
|  | Place the baby on a firm warm surface or under a radiant heat warmer? | 1 | 0 | 8 |
|  | Position baby’s head in a neutral position (slightly extended position) to open airway (as shown below)?   |  |  |  |
|  | Open airway | 1 | 0 | 8 |
|  | Assess breathing. | 1 | 0 | 8 |
|  | Able to recognize any of the three scenarios (i.e. No breathing, Gasping or Normal breathing) occurring to the baby? | 1 | 0 | 8 |
|  | Recheck the newborn’s position, if gasping or no breathing and place the mask on the newborn’s face – covering the chin, mouth and nose? | 1 | 0 | 8 |
|  | Chest compression | 1 | 0 | 8 |
| **18** | **Neonatal infections**  **(Circle: YES if neonate has this condition; and NO if not)**  Which infections or condition does the provider say the baby has? |  |  |  |
|  | 1. Skin infections | 1 | 0 | 8 |
|  | 1. Eye infection | 1 | 0 | 8 |
|  | 1. Oral thrush | 1 | 0 | 8 |
|  | 1. Cord infection | 1 | 0 | 8 |
|  | 1. Septicemia | 1 | 0 | 8 |
|  | 1. Neonatal Tetanus | 1 | 0 | 8 |
|  | 1. Jaundice | 1 | 0 | 8 |
| **18.1** | **For skin infections, does the provider:** |  |  |  |
|  | 1. Wash hands with soap and water before and after handling baby? | 1 | 0 | 8 |
|  | 1. Clean skin with antiseptic lotions like Hibitane? | 1 | 0 | 8 |
|  | 1. Give or prescribe appropriate antibiotics? | 1 | 0 | 8 |
|  | 1. Counsel the mother on subsequent care? | 1 | 0 | 8 |
| **18.2** | **For neonatal eye infection, does the provider:** |  |  |  |
|  | 1. Observe infection prevention practices which include washing hands before and after procedure? | 1 | 0 | 8 |
|  | 1. Clean baby’s eyes with normal saline before administering medication? | 1 | 0 | 8 |
|  | 1. Administer appropriate antibiotic eye ointment? | 1 | 0 | 8 |
| **18.3** | **For Oral thrush, does the provider:** |  |  |  |
|  | 1. Wash hands | 1 | 0 | 8 |
|  | 1. Clean baby’s mouth with a clean soft cloth | 1 | 0 | 8 |
|  | 1. Instill Nystatin drops and give instructions on administration? | 1 | 0 | 8 |
|  | 1. Continue breast feeding | 1 | 0 | 8 |
|  | 1. Treat mother’s breast with the same medicine | 1 | 0 | 8 |
| **18.4** | **For Cord Infection, does the provider:** |  |  |  |
|  | 1. Wash hands before handling the cord | 1 | 0 | 8 |
|  | 1. Wear clean gloves | 1 | 0 | 8 |
|  | 1. Clean the cord with antiseptic solution e.g. povidine (tincture) iodine with clean gauze/ cotton wool | 1 | 0 | 8 |
|  | 1. Apply Gentian Violet four times a day | 1 | 0 | 8 |
|  | 1. Keep cord dry | 1 | 0 | 8 |
|  | 1. Keep baby clean | 1 | 0 | 8 |
|  | 1. Continue breast feeding | 1 | 0 | 8 |
|  | 1. Give Amoxicillin 62.5g mg/kg – three times a day for 5 days | 1 | 0 | 8 |
|  | 1. Admit or refer baby for admission if signs are severe | 1 | 0 | 8 |
|  | 1. Others (specify) |  |  |  |
| **18.5** | **For Neonatal septicaemia, does the provider:** | 1 | 0 | 8 |
|  | 1. Give pre-referral treatment (IV Crystalline Penicillin and Gentamicin) | 1 | 0 | 8 |
|  | 1. Keep baby warm | 1 | 0 | 8 |
|  | 1. Prevent hypoglycemia by feeding the baby (breast feeding/ Expressed Breast Milk) | 1 | 0 | 8 |
|  | 1. Others (Specify cause of septicaemia)…………………… | 1 | 0 | 8 |
| **18.6** | **For Neonatal Tetanus, does the provider ascertain:** |  |  |  |
|  | 1. TT immunization status of the mother | 1 | 0 | 8 |
|  | 1. Date of delivery | 1 | 0 | 8 |
|  | 1. Stiffness of neonate | 1 | 0 | 8 |
|  | 1. General condition | 1 | 0 | 8 |
|  | 1. Pulse, temperature, respiration | 1 | 0 | 8 |
|  | 1. Ability to suck (breastfeeding) | 1 | 0 | 8 |
|  | 1. Ability to open mouth | 1 | 0 | 8 |
|  | 1. Does the provider admit or refer the baby for admission? | 1 | 0 | 8 |
| **19** | **Observation of Low birth weight neonate/Premature babies**  (This could be any baby whose birth weight is below 2500gms at birth. Or very low birth weight infant (any baby whose birth weight is below 1500gms at birth). Or pre-term baby (Any baby born before 37 completed weeks of gestation).  **(Circle: YES if neonate has this condition; and NO if not)**  Does the provider: |  |  |  |
|  | 1. Take history to determine maturity by dates | 1 | 0 | 8 |
|  | 1. Conduct neonatal assessment to determine gestational age clinically | 1 | 0 | 8 |
|  | 1. Admit the baby in the newborn unit | 1 | 0 | 8 |
|  | 1. Transfer or recommend the transfer of the baby immediately to a centre with a newborn unit | 1 | 0 | 8 |
|  | 1. Provide adequate warmth (or discuss with the mother on how to provide warmth e.g. through KMC approach) | 1 | 0 | 8 |
|  | 1. How to express milk | 1 | 0 | 8 |
|  | 1. Explain how feeding will be done given the baby’s condition | 1 | 0 | 8 |
|  | 1. How to prevent infections | 1 | 0 | 8 |
|  | **For Neonatal Jaundice (**yellow discoloration of the skin and mucous membranes as a result of raised bilirubin levels occurring in the first 28 days of life).  **Does the provider:** |  |  |  |
|  | 1. Take history to determine the cause of jaundice? | 1 | 0 | 8 |
|  | 1. Check on the following during examination? | 1 | 0 | 8 |
|  | - 1. Yellowness of skin and mucus membranes | 1 | 0 | 8 |
|  | - 1. Colour of urine and stools | 1 | 0 | 8 |
|  | - 1. General behavior and activity | 1 | 0 | 8 |
|  | - 1. Signs of infection | 1 | 0 | 8 |
|  | - 1. Ability to suck properly | 1 | 0 | 8 |
|  | - 1. Check for pallor | 1 | 0 | 8 |
|  | - 1. Monitor for signs of kernicterus and act promptly | 1 | 0 | 8 |
|  | - 1. Monitor bilirubin levels | 1 | 0 | 8 |
|  | - 1. Inform the mother about the results of the bilirubin levels | 1 | 0 | 8 |
|  | - 1. Manage the baby as per the results of the bilirubin levels? | 1 | 0 | 8 |
|  | 1. Refer for investigations | 1 | 0 | 8 |

|  | **Neonatal Emergencies: Other Common Conditions** |  |  |  |
| --- | --- | --- | --- | --- |
| **21.1** | **Respiratory Distress** (a baby has difficulty in breathing).  **(Circle: YES if neonate has this condition; and NO if not)**  Does the provider: |  |  |  |
|  | 1. Establish that the baby has signs and symptoms of Respiratory Distress e.g. a fast breathing rate, Chest in-drawing, nasal flaring, grunting? | 1 | 0 | 8 |
|  | 1. Keep the baby warm | 1 | 0 | 8 |
|  | 1. Give oxygen | 1 | 0 | 8 |
|  | 1. Give antibiotics – Crystalline Penicillin and Gentamicin | 1 | 0 | 8 |
|  | 1. Feed the neonate | 1 | 0 | 8 |
|  | 1. Give vitamin K | 1 | 0 | 8 |
|  | 1. Admit or refer | 1 | 0 | 8 |
| **21.2** | **Neonatal Apnoea {**This is cessation of spontaneous respirations (>20 seconds) accompanied by bradycardia and cyanosis}.  **(Circle: YES if neonate has this condition; and NO if not)**  Does the provider: |  |  |  |
|  | 1. Make the diagnosis on the basis of: |  |  |  |
|  | - Absence of respirations lasting 20 seconds or more | 1 | 0 | 8 |
|  | - Cyanosis | 1 | 0 | 8 |
|  | - Heart rate below 100/minute | 1 | 0 | 8 |
|  | 1. Keep baby warm | 1 | 0 | 8 |
|  | 1. Stimulate baby by stroking/rubbing (do not slap) soles of the feet | 1 | 0 | 8 |
|  | 1. Position baby to open airway and Clear airways if necessary | 1 | 0 | 8 |
|  | 1. If still not breathing give oxygen and assist ventilation | 1 | 0 | 8 |
|  | 1. Investigate to establish cause; ***Treat cause if known*** | 1 | 0 | 8 |
|  | 1. Stop oral feeds and give intravenous fluid (IV) until condition improves | 1 | 0 | 8 |
| **21.3** | **Convulsions (Neonatal seizures).** These are involuntary movements of one or more parts of the body.  **(Circle: YES if neonate has this condition; and NO if not)**  Does the provider: |  |  |  |
|  | 1. Make the diagnosis on the basis of: |  |  |  |
|  | - Maternal history of difficult delivery, or infection | 1 | 0 | 8 |
|  | - Perinatal asphyxia | 1 | 0 | 8 |
|  | - History from the mother of abnormal movements | 1 | 0 | 8 |
|  | 1. Give emergency treatment as follows: |  |  |  |
|  | - Emergency treatment should begin immediately irrespective of the cause | 1 | 0 | 8 |
|  | - Support vital functions e.g. position well to ensure patent air-way | 1 | 0 | 8 |
|  | - Give oxygen | 1 | 0 | 8 |
|  | - Give anticonvulsant e.g. IM Phenobarbitone | 1 | 0 | 8 |
|  | - Give 2ml/kg IV 10% dextrose solution as bolus | 1 | 0 | 8 |
|  | - Keep the baby warm | 1 | 0 | 8 |
|  | - Tell the mother to initiate/resume breastfeeding or give expressed breast milk | 1 | 0 | 8 |
|  | - Refer after stabilization to newborn unit if cannot manage | 1 | 0 | 8 |
| **21.4** | **Neonatal Hypoglycaemia (**This occurs when the blood glucose level is below 2.6 mmol /l (45 mg/dl) irrespective of gestation and postnatal age).  **(Circle: YES if neonate has this condition; and NO if not)**  Does the provider: |  |  |  |
|  | 1. Assess for features such as jitteriness, sweating, convulsions, apnoea, cyanosis, hypotonia | 1 | 0 | 8 |
|  | 1. Make a distinction on how to manage the baby if the blood glucose is less than 1.1 mmol /l (25 mg/dl) versus when the blood glucose is between 1.1 -2.6m/mol/l (25-45mg/dl) | 1 | 0 | 8 |
| **21.5** | **Hypothermia (**This is a condition where the baby’s temperature falls below 36.5o C (based on axillary temperature)  **(Circle: YES if neonate has this condition; and NO if not)**  Does the provider: |  |  |  |
|  | 1. Classify hypothermia into: |  |  |  |
|  | 1. Low body temperature 35.5oC to 36.40C | 1 | 0 | 8 |
|  | 1. Very low temperature less than 35.50C | 1 | 0 | 8 |
|  | 1. Keep the baby warm by: |  |  |  |
|  | 1. Removing wet/cold clothes | 1 | 0 | 8 |
|  | 1. Skin-to-skin contact with the mother and cover with warm linen | 1 | 0 | 8 |
|  | 1. Adequately clothing the baby (including hat and socks) | 1 | 0 | 8 |
|  | 1. Keeping clothed baby under radiant heat source; Nursing in a warm incubator if possible | 1 | 0 | 8 |
|  | 1. Giving oxygen if baby is blue or having difficulty in breathing | 1 | 0 | 8 |
|  | 1. Passing nasogastric tube and giving breast milk | 1 | 0 | 8 |
|  | 1. Re-checking the temperature after one hour and repeating hourly until it reaches the normal range (36.50C -37.40C) | 1 | 0 | 8 |
| **21.6** | **Bleeding in the Newborn** (This is when a baby presents with bleeding)  **(Circle: YES if neonate has this condition; and NO if not)**  Does the provider: |  |  |  |
|  | 1. Take history and Examine baby? | 1 | 0 | 8 |
|  | 1. Ensure warmth? | 1 | 0 | 8 |
|  | 1. Investigate to identify cause? | 1 | 0 | 8 |
|  | 1. Treat the cause immediately? | 1 | 0 | 8 |
|  | - If from cord stump, re-tie or re-clamp | 1 | 0 | 8 |
|  | - If cut, press on bleeding site with sterile gauze | 1 | 0 | 8 |
|  | 1. Give Vitamin K 1 mg/kg IV even if the baby had already been given? | 1 | 0 | 8 |
|  | 1. Transfuse if the signs of shock are present and also give oxygen? | 1 | 0 | 8 |
| **21.7** | **Hyperthermia (**a condition where the baby’s temperature is above 37.5^o^C)  **(Circle: YES if neonate has this condition; and NO if not)**  Does the provider: |  |  |  |
|  | Expose the baby? | 1 | 0 | 8 |
|  | Identify the cause? | 1 | 0 | 8 |
|  | Treat the cause? | 1 | 0 | 8 |

|  | **Birth Injuries**  **(Circle: YES if neonate has this condition; and NO if not)** |  |  |  |
| --- | --- | --- | --- | --- |
|  | Does the provider proactively look for and assess birth injuries? | 1 | 0 | 8 |
|  | Does the provider mention any common injury she or he is looking out for? | 1 | 0 | 8 |
|  | If Yes, which one or which ones are mentioned? | 1 | 0 | 8 |
|  | 1. Skin and superficial tissue injuries on presenting parts (e.g. head, face, genitalia etc.) | 1 | 0 | 8 |
|  | 1. Muscle trauma | 1 | 0 | 8 |
|  | 1. Nerve trauma | 1 | 0 | 8 |
|  | 1. Fractures | 1 | 0 | 8 |
|  | 1. Others (specify) | 1 | 0 | 8 |
|  | **Congenital Anomalies**  **(Circle: YES if neonate has this condition; and NO if not)**  Does the provider: |  |  |  |
|  | 1. Identify the nature of congenital abnormality? | 1 | 0 | 8 |
|  | 1. Stabilize the baby | 1 | 0 | 8 |
|  | 1. Refer or recommend the transfer of the baby immediately to the nearest   Hospital capable of dealing with the problem? | 1 | 0 | 8 |
|  | 1. Counsel parents or have the most qualified health worker talk to parents? | 1 | 0 | 8 |
|  | 1. Show parents obvious defect on the baby and told the implications? | 1 | 0 | 8 |
|  | **Large for Gestational age** (This is a baby with a birth weight of more than 4.0kg; **OR** A baby whose birth weight is above the 90th percentile for the gestation).  **(Circle: YES if neonate has this condition; and NO if not)**  Does the provider: |  |  |  |
|  | 1. Find out about the history of diabetes in pregnancy or history of previous large babies in the family? | 1 | 0 | 8 |
|  | 1. Initiate breastfeeding immediately and continue feeding on demand? | 1 | 0 | 8 |
|  | 1. Closely monitor the baby to promptly recognize the associated problems | 1 | 0 | 8 |
|  | 1. Manage any complications detected | 1 | 0 | 8 |
|  | 1. Test or request for the testing of blood sugar levels where possible | 1 | 0 | 8 |
|  | 1. Keep the baby warm | 1 | 0 | 8 |

| 1. **ADDITIONAL ISSUES TO BE OBSERVED AND SUMMARY IMPRESSIONS** | | | | |
| --- | --- | --- | --- | --- |
| 25.1 | DOES THE CLIENT/MOTHER ASK THE PROVIDER QUESTIONS? | **YES** | **NO** | **N/A** |
|  |  | 1 | 0 | 8 |
| 25.2 | *DOES THE PROVIDER:* |  |  |  |
| a) | Use client’s name when talking to him/her | 1 | 0 | 8 |
| b) | Listen to the client | 1 | 0 | 8 |
| c) | Ask questions | 1 | 0 | 8 |
| d) | Help in decision making | 1 | 0 | 8 |
| e) | Ask if client understood the information | 1 | 0 | 8 |
| f) | Encourage client to ask questions | 1 | 0 | 8 |
| g) | Use client record | 1 | 0 | 8 |
| i) | Ask tactfully but clearly about client’s concerns and answer honestly, directly, and without embarrassment. | 1 | 0 | 8 |
| j) | Ask for clarification, if needed, and check understanding. | 1 | 0 | 8 |
| k) | Avoid expressing judgment (or being judgemental). | 1 | 0 | 8 |
| l) | Show attitude favourable to offering quality neonatal care (e.g. requests the client to ask for points of clarification or to share his/her concerns regarding other medical conditions the baby could be having) | 1 | 0 | 8 |

| **TIME OBSERVATION ENDED (24 HRS): HOUR MINUTE** |
| --- |
