# Supplementary material for: Evaluating quality neonatal care, call Centre service, tele-health and community engagement in reducing newborn morbidity and mortality in Bungoma county, Kenya
Source: BMC Health Serv Res. 2018 Jun 25;18:493. doi: 10.1186/s12913-018-3293-5 (PMC6019716; doi:10.1186/s12913-018-3293-5)
Supplement: Supplementary file 3 — Tool for health provider’s interview. (DOCX 67 kb) [file 12913_2018_3293_MOESM3_ESM.docx]

**INTERVIEW WITH A HEALTH PROVIDER**

| **SERIAL NUMBER** | ___\| ___\| ___\| |
| --- | --- |

**COLLABORATIVE NEW BORN SUPPORT PROJECT**

1. Facility Name ______________________________ Code_________(Insert Code from the list below)

| **Health Facility** | **Code** | **Health Facility** | **Code** |
| --- | --- | --- | --- |
| Webuye county Hospital |  | Kapsakwony sub-county hospital | 06 |
| Sirisia Sub- County Hospital |  | Bumula sub-county hospital | 07 |
| Kimilili Sub County hospital |  | Chwele sub County Hospitals. | 08 |
| Naitiri Sub county Hospital |  | Sinoko Sub-County Hospital | 09 |
| Bungoma County Referral Hosp. |  |  |  |

1. Sub-County name for selected facility …………………………… Code_________(Insert Code from list below)

| **Name of Sub-County** | **Code** | **Name of Sub-County** | **Code** |
| --- | --- | --- | --- |
| Bumula |  | Tongaren | (6) |
| Kanduyi |  | Mt. Elgon | (7) |
| Kabuchai |  | Webuye East | (8) |
| Sirisia |  | Webuye West | (9) |
| Kimilili |  |  |  |

## Status of the selected hospital: Circle appropriate code: Phase I =01; Phase II =02

1. Section/Unit where Health Provider works (Circle appropriate box)*.*

| Nursery | 01 | Maternity/Delivery Room | 02 | MCH | 03 | Post Natal ward | 04 | Paediatrics ward | 05 | OPD | 06 |
| --- | --- | --- | --- | --- | --- | --- | --- | --- | --- | --- | --- |

1. Date of Interview (DAY, MONTH, YEAR E.G. 02/03/16) [___|___/___|___/___|___]

| 1. Interview Result | Completed | 01 |
| --- | --- | --- |
|  | Partially Completed | 02 |
|  | Refused | 03 |
|  | Other (specify)________________ | 96 |

1. Name of Interviewer ………………………………………………………………... Signature ………………………………..
2. Supervisor’s Name ………………………………………………….…….…………..… Signature ……………………………….

| **Section 1: Background** | | | | | | | | | | | | |
| --- | --- | --- | --- | --- | --- | --- | --- | --- | --- | --- | --- | --- |
| **NO.** | **QUESTION** | | **RESPONSE OPTIONS** | | | | **CODES** | | | | **SKIP** | |
|  | Provider is... | | Female | | | | 1 | | | |  | |
|  |  |  | Male | | | | 2 | | | |  |  |
|  | How old are you? | | Age (years) | | | | _______ | | | |  | |
|  |  |  | Don’t know | | | | 98 | | | |  |  |
|  | What is your professional qualification?  **[DO NOT READ OPTIONS]** | | Medical Officer | | | | 1 | | | |  | |
|  |  |  | Paediatrician | | | | 2 | | | |  |  |
|  |  |  | Obstetrician/Gynaecologist | | | | 3 | | | |  |  |
|  |  |  | Clinical officer | | | | 4 | | | |  |  |
|  |  |  | Clinical Paediatrics | | | | 5 | | | |  |  |
|  |  |  | Enrolled nurse/midwife | | | | 6 | | | |  |  |
|  |  |  | Registered nurse/midwife | | | | 7 | | | |  |  |
|  |  |  | BSc nurse | | | | 8 | | | |  |  |
|  |  |  | Other (specify)______________ | | | | 88 | | | |  |  |
|  | For how many years have you worked in...?  **[READ OUT OPTIONS. RECORD ’00’ IF LESS THAN ONE YEAR]** | |  | | | **Years** | **Don’t remember** | | | |  | |
|  |  |  | a) Health care | | | _________ | 998 | | | |  |  |
|  |  |  | b) This facility | | | _________ | 998 | | | |  |  |
|  |  |  | c) This unit | | | _________ | 998 | | | |  |  |
|  | *In a typical week,* approximately how many newborn babies do you provide with care or services? | | | | | | No----------------- | | | | | |
|  | During the **last one year**, have you received in-service training in the following areas? (**READ OUT & CIRCLE ‘1’ FOR ‘YES’ TO ALL THAT APPLY]** | | | | | | **Yes** | | **No** | **Don't Know** | |  |
|  | a) Family planning | | | | | | 1 | | 0 | 98 | |  |
|  | b) Contraceptive technology update | | | | | | 1 | | 0 | 98 | |  |
|  | c) Focused antenatal care (FANC) | | | | | | 1 | | 0 | 98 | |  |
|  | d) Targeted postnatal care | | | | | | 1 | | 0 | 98 | |  |
|  | e) Syndromic diagnosis and management of STIs | | | | | | 1 | | 0 | 98 | |  |
|  | f) Prevention of mother-to-child transmission of HIV/AIDS (PMTCT) | | | | | | 1 | | 0 | 98 | |  |
|  | g) Voluntary counselling and testing for HIV/AIDS | | | | | | 1 | | 0 | 98 | |  |
|  | j) ART/management of HIV/AIDS infected clients | | | | | | 1 | | 0 | 98 | |  |
|  | k) Screening for cervical cancer (VIA/VILLI) | | | | | | 1 | | 0 | 98 | |  |
|  | l) Integration of counselling and testing for HIV into FP | | | | | | 1 | | 0 | 98 | |  |
|  | m) A. Newborn care | | | | | | 1 | | 0 | 98 | |  |
|  | m) B. If yes in m (A), specify type of newborn in-service training course received ………………………………………………………………………………………… | | | | | |  | |  |  | |  |
|  | n) the new Harmonised EmONC curriculum | | | | | | 1 | | 0 | 98 | |  |
|  | o) Care of the sick newborn | | | | | | 1 | | 0 | 98 | |  |
|  | s) Infection control | | | | | | 1 | | 0 | 98 | |  |
|  | t)Maternal and Perinatal Death Review, Surveillance and Response | | | | | | 1 | | 0 | 98 | |  |
|  | v) Infection control (including post exposure prophylaxis) | | | | | | 1 | | 0 | 98 | |  |
|  | v) Other (specify)_____________________________________ | | | | | | 1 | | 0 | 98 | |  |
|  | How do you refer clients seeking various RH services to other units such as MCH/FP, EPI, maternity, laboratory clinic?  **[DO NOT READ LIST. CIRCLE ‘1’ FOR ‘YES’ TO ALL THAT APPLY AND PROBE BY ASKING ‘ANY OTHER’]** | | | | a) Referral slips | | 1 | | 0 | 98 | |  |
|  |  |  |  |  | b) Registers | | 1 | | 0 | 98 | |  |
|  |  |  |  |  | c) Information recorded on clients’ card | | 1 | | 0 | 98 | |  |
|  |  |  |  |  | d) Tell client but do not write anywhere | | 1 | | 0 | 98 | |  |
|  |  |  |  |  | e) Escort client to clinic | | 1 | | 0 | 98 | |  |
|  |  |  |  |  | f) None | | 1 | | 0 | 98 | |  |
|  |  |  |  |  | g) Other (specify) _____________ | | 1 | | 0 | 98 | |  |
|  | How do you refer clients seeking various newborn services to other health facilities?  **[DO NOT READ LIST. CIRCLE ‘1’ FOR ‘YES’ TO ALL THAT APPLY AND PROBE BY ASKING ‘ANY OTHER’]** | | | | a) Referral slips | | 1 | | 0 | 98 | |  |
|  |  |  |  |  | b) Registers | | 1 | | 0 | 98 | |  |
|  |  |  |  |  | c) Information recorded on clients’ card | | 1 | | 0 | 98 | |  |
|  |  |  |  |  | d) Tell client but do not write anywhere | | 1 | | 0 | 98 | |  |
|  |  |  |  |  | e) Escort client | | 1 | | 0 | 98 | |  |
|  |  |  |  |  | f) None | | 1 | | 0 | 98 | |  |
|  |  |  |  |  | g) Other (specify) _____________ | | 1 | | 0 | 98 | |  |
|  | How are clients seeking neonatal services referred to another facility/ clinic from the community level?  **[DO NOT READ LIST. CIRCLE ‘1’ FOR ‘YES’ TO ALL THAT APPLY AND PROBE BY ASKING ‘ANY OTHER’]** | | | | a) Referral slips | | 1 | | 0 | 98 | |  |
|  |  |  |  |  | b) Registers | | 1 | | 0 | 98 | |  |
|  |  |  |  |  | c) Information recorded on clients’ card | | 1 | | 0 | 98 | |  |
|  |  |  |  |  | d) Tell client but do not write anywhere | | 1 | | 0 | 98 | |  |
|  |  |  |  |  | e) Escort client to clinic | | 1 | | 0 | 98 | |  |
|  |  |  |  |  | f) None | | 1 | | 0 | 98 | |  |
|  |  |  |  |  | g)Other (specify)________________ | | 1 | | 0 | 98 | |  |
| **Section 2: Integration of newborn and other RH Services** | | | | | | | | | | | | |
|  | | What services do you personally provide at this facility?  *Interviewer: Mark all that apply.* | | FP counseling | | | |  | | | | |
|  |  |  |  | Contraceptives | | | |  | | | | |
|  |  |  |  | ANC | | | |  | | | | |
|  |  |  |  | Delivery | | | |  | | | | |
|  |  |  |  | Postnatal care | | | |  | | | | |
|  |  |  |  | New born care | | | |  | | | | |
|  |  |  |  | Management of the sick neonate | | | |  | | | | |
|  |  |  |  | Treatment of abortion complications | | | |  | | | | |
|  |  |  |  | HIV/AIDS counseling /testing | | | |  | | | | |
|  |  |  |  | HIV/AIDS treatment and care | | | |  | | | | |
|  |  |  |  | EmONC | | | |  | | | | |
|  |  |  |  | STI services | | | |  | | | | |
|  |  |  |  | Child immunization | | | |  | | | | |
|  |  |  |  | Child growth monitoring | | | |  | | | | |
|  |  |  |  | Curative services adults | | | |  | | | | |
|  |  |  |  | Curative services for children | | | |  | | | | |
|  |  |  |  | Other (SPECIFY-----------): | | | |  | | | | |

**Section 3: Key Elements for Newborn Care**

The following table contains key elements that are important for the care of Newborns.

|  |  |  | Yes | No | N/A | | |
| --- | --- | --- | --- | --- | --- | --- | --- |
|  | Availability of Policies | 1. Are there service-delivery guidelines (SDGs) for the care of Newborns? | 1 | 0 | 8 | | |
|  |  | 1. If yes, do you have access to these service-delivery guidelines (SDGs) for the care of Newborns? | 1 | 0 | 8 | | |
|  | Information, Education and Communication/BCC | 1. Are there client’s brochures or posters in the facility that address the care of Newborns? | 1 | 0 | 8 | | |
|  |  | 1. If yes, do the facility’s brochures or posters visibly reflect for the care of Newborns? | 1 | 0 | 8 | | |
|  |  | 1. Do you have IEC materials that target community members? | 1 | 0 | 8 | | |
|  | Counseling | Do providers protect the client’s right to privacy during counseling procedures and service delivery? | 1 | 0 | 8 | | |
|  | Referral | Is there an effective referral mechanism:  Within the community level (e.g. CHV to CM or to the CHEW)  From the Community to Facility,  Intra-facility/Within facility  Inter-facility/between facilities (from lower to higher ones) |  | | | |  |
|  |  |  | 1 | 0 | 8 | | |
|  |  |  | 1 | 0 | 8 | | |
|  |  |  | 1 | 0 | 8 | | |
|  | Drugs, Supplies | Would you say that the drugs and supplies for new-born care –in this facility are available most of the time? | 1 | 0 | 8 | | |
|  | Community Partnerships and Resources | Does this facility have outreach programmes through which it links with communities or villages to facilitate service delivery to women and their newborn babies? | 1 | 0 | 8 | | |
|  | **Linkages and Access:** |  |  |  |  | | |
| i) | How are CHVs & CMs) linked to this health facility? | 1. No, they are not linked | 0 | | |  |  |
|  |  | 1. Through periodic regular meetings | 1 | | |  |  |
|  |  | 1. During training sessions | 2 | | |  |  |
|  |  | 1. During referral of clients/ CHVs and CMs accompany referred clients/patients | 3 | | |  |  |
|  |  | 1. Others (specify) | 88 | | |  |  |
| ii) | On average, how long does it take the health facility staff to respond or act on requests for help in case of emergencies made by CHVs and CMs? | 1. CMs and CHVs do not usually make any requests | 0 | | |  |  |
|  |  | 1. Staff in the health facility do not act at all | 1 | | |  |  |
|  |  | 1. Immediately (i.e. within hours) | 2 | | |  |  |
|  |  | 1. Takes one day | 3 | | |  |  |
|  |  | 1. Takes 2-4 days | 4 | | |  |  |
|  |  | 1. Over 5 days | 5 | | |  |  |
|  |  | 1. Depends on the nature of the request or issue and whether it is an emergency or not | 6 | | |  |  |
|  |  | 1. Others (specify) | 88 | | |  |  |

|  | What does the basic preventive care for newborn include?  **[DO NOT READ LIST. CIRCLE ‘1’ FOR ‘YES’ TO ALL THAT APPLY AND PROBE BY ASKING ‘ANY OTHER’]** |  | **Yes** | **No** |  |
| --- | --- | --- | --- | --- | --- |
|  |  | a) Exclusive breastfeeding for 6 months | 1 | 0 |  |
|  |  | b) Early detection of problems and danger signs | 1 | 0 |  |
|  |  | c) Timely treatment of complications | 1 | 0 |  |
|  |  | d) Clean delivery practices | 1 | 0 |  |
|  |  | e) Warmth | 1 | 0 |  |
|  |  | f) Cord care | 1 | 0 |  |
|  |  | g) Eye care | 1 | 0 |  |
|  |  | h) Early initiation of breastfeeding | 1 | 0 |  |
|  |  | i) Vaccination/immunization |  |  |  |
|  |  | j) Other (specify) _______________ | 1 | 0 |  |
|  | What are the danger signs and symptoms of infection in the newborn?  **[DO NOT READ LIST. CIRCLE ‘1’ FOR ‘YES’ TO ALL THAT APPLY AND PROBE BY ASKING ‘ANY OTHER’]** |  | **Yes** | **No** |  |
|  |  | a) Poor or no breastfeeding /feeding | 1 | 0 |  |
|  |  | b) Difficulty breathing | 1 | 0 |  |
|  |  | c) Hypothermia or hyperthermia | 1 | 0 |  |
|  |  | d) Septic spots /boils on body | 1 | 0 |  |
|  |  | e) Restlessness or irritability | 1 | 0 |  |
|  |  | f) Jaundice at birth | 1 | 0 |  |
|  |  | g)Foul smelling discharge from the umbilical cord |  |  |  |
|  |  | h) Other (specify)  __________________________________ | 1 | 0 |  |
|  | What kind of messages would you specifically give to HIV positive mothers to promote safe infant feeding?  **[DO NOT READ LIST. CIRCLE ‘1’ FOR ‘YES’ TO ALL THAT APPLY AND PROBE BY ASKING ‘ANY OTHER’]** |  | **Yes** | **No** |  |
|  |  | a) Exclusive breast feeding (6 months) | 1 | 0 |  |
|  |  | b) Exclusive replacement feeding | 1 | 0 |  |
|  |  | c) No mixed feeding | 1 | 0 |  |
|  |  | d) Other (specify)  __________________________________ | 1 | 0 |  |

**Section 4: Self-reported provider knowledge and skills Care**

|  | **On a scale of 1 = (poor), 2 = (satisfactory/fair) and 3= (good/excellent); how would you rate your own skills and knowledge in managing the following neonatal diseases and conditions?** | | | |
| --- | --- | --- | --- | --- |
|  | **Disease/Condition** | **Poor** | **(satisfactory/fair)** | **good/excellent** |
|  | Care of the normal neonate | 1 | 2 | 3 |
|  | Breastfeeding | 1 | 2 | 3 |
|  | Resuscitation of the neonate | 1 | 2 | 3 |
|  | Birth injuries | 1 | 2 | 3 |
|  | **Management of high risk neonate** | 1 | 2 | 3 |
|  | Infection prevention in the newborn | 1 | 2 | 3 |
|  | Low Birth Weight | 1 | 2 | 3 |
|  | Anemia of prematurity | 1 | 2 | 3 |
|  | Congenital/ perinatal infections | 1 | 2 | 3 |
|  | **Neonatal Emergencies** | 1 | 2 | 3 |
|  | Respiratory Distress | 1 | 2 | 3 |
|  | Neonatal Apnoea | 1 | 2 | 3 |
|  | Convulsions (neonatal seizures) | 1 | 2 | 3 |
|  | Neonatal Hypoglycemia | 1 | 2 | 3 |
|  | Hypothermia | 1 | 2 | 3 |
|  | Bleeding in the newborn | 1 | 2 | 3 |
|  | Fluid management (neonatal) | 1 | 2 | 3 |
|  | Neonatal infections | 1 | 2 | 3 |
|  | Neonatal jaundice | 1 | 2 | 3 |
|  | Please state one main reason why you felt that your knowledge and skills for managing certain conditions are poor.  --------------------------------------------- |  |  |  |

|  | What are the main challenges you face in providing neonatal care services in this facility and within the local community? (Circle the codes) | Lack of supplies | 1 |  |
| --- | --- | --- | --- | --- |
|  |  | Lack of qualified personnel | 2 |  |
|  |  | Lack of equipment | 3 |  |
|  |  | Failures in equipment | 4 |  |
|  |  | Inappropriate facilities | 5 |  |
|  |  | Do not feel sufficiently trained | 6 |  |
|  |  | Not enough time to counsel or manage newborn care services | 7 |  |
|  |  | No challenge faced | 8 |  |
|  |  | Other (specify): | 9 |  |
| \| **32** \| ANY OTHER COMMENTS/IMPRESSIONS (WRITE OVERLEAF IF NECESSARY) \| \| --- \| --- \| \| TIME ENDED: HOUR MINUTE \| | | | | |
