# Supplementary material for: Evaluating quality neonatal care, call Centre service, tele-health and community engagement in reducing newborn morbidity and mortality in Bungoma county, Kenya
Source: BMC Health Serv Res. 2018 Jun 25;18:493. doi: 10.1186/s12913-018-3293-5 (PMC6019716; doi:10.1186/s12913-018-3293-5)
Supplement: Supplementary file 4 — Guide for key informant interviews for health managers. (DOCX 56 kb) [file 12913_2018_3293_MOESM4_ESM.docx]

**Assessing the Capacity of Bungoma County and Sub-county Health facilities to provide new born services through the Collaborative New born Support Project**

## Background Details of Key informants

**Name of County ___________________________________**

**INTERVIEWER: _____________________Code**

**Note taker: ______________________Code
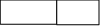
**

**Sub-County _______________________ Code
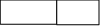
**

**Tick the respondent’s responsibility and title team member:**

County HMT Sub-County HMT Facility in charge

Maternity in Charge NBU in Charge PNC in Charge

**For facility in-charges fill the following**

1. Name of Heath Facility ____________________________________
2. Facility Ownership:

Government Private FBO NGO

1. Type of facility

County Referral Hospital 01

Private Hospital 02

Sub County Hospital 03

Health Centre 04

Dispensary 05

Clinic 06

Other specify ___________________

**DATE OF INTERVIEW (dd-mm-yy):
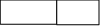

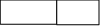

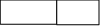
**

**TIME STARTED (24 hours) (hr-mm):
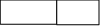

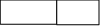
**

**TIME ENDED: (24 hours) (hr-mm):
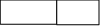

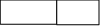
**

**Key Informant interview guide for health managers**

1. **Description of key informant’s role in leadership and governance in the provision of new born health**
   1. Can you please explain to me your position in this health facility?
   2. What is your role in the provision of new born health services? [***Probe*** *for involvement in management or implementation role in new-born care*].
   3. Any specific roles in the management of:
2. *Normal new-born babies?*
3. *Sick new-born babies requiring resuscitation or emergency treatment and or admission/referral?*
   1. How are services for new-born babies supervised? Who supervises whom? How frequent?
   2. How do you guarantee the provision of quality new-born care services?
   3. Do you have specific strategies for creating demand for various new-born care services within the catchment area? If Yes-please elaborate on these strategies
4. **Understanding of services offered in the new born unit**
5. What new born services are offered in this hospital? **Probe** for the services offered in ANC, labour, delivery, PNC including management of neonatal complications
6. In your own opinion, please describe the quality of care of the services offered by the current new born unit? *PROBE for description of services provided, work load, responsibilities, infection prevention practices*, *referral procedures*
7. Are there examples of projects where this hospital has in the past partnered with the local community to improve the health status of women and new-borns?
8. In general, what do you think should constitute quality of care in the New Born Unit? *PROBE for description of services provided, infection prevention practices*
9. To what extent are service delivery guidelines for new-born care used by health providers?
10. Explore current obstacles to accessing new born care such as unavailability of 24 hours service or closure during weekends, poor skills, socio-cultural barriers etc.
11. What additional new born care services should hospital provide?
12. There is need to have facilities with varying functional capabilities for providing inpatient care for newborn infants. Four levels of Neonatal Care (based on functional capabilities) are described below. Listen very carefully to the description so that you may offer guidance to the discussions that will follow thereafter.

***Level I (basic):*** a hospital nursery organized with the personnel and equipment to perform neonatal resuscitation, evaluate and provide postnatal care of healthy newborn infants, stabilize and provide care for infants born at 35 to 37 weeks' gestation who remain stable.

***Level II (specialty Care):*** a hospital special care nursery organized with the personnel (such as paediatricians, specialized nurses, clinical officers) and equipment to provide care to infants born at more than 32 weeks' gestation and weighing more than 1500 g who have physiologic immaturity, inability to maintain body temperature, or inability to take oral feedings; who are moderately ill or who are convalescing from intensive care.

***Level III (subspecialty Care)****:* a hospital Newborn Intensive Care Unit organized with personnel and equipment to provide continuous life support and comprehensive care for extremely high-risk newborn infants (<32 wks and <1500 g) and all critically ill infants and those with complex illness. The hospital provides a full range of respiratory support and has readily available paediatric medical subspecialists and paediatric surgical specialists.

***Level IV (Subspecialty Care)*:** Located within an institution with the capability to provide surgical repair of complex congenital or acquired conditions. Immediate on-site access to pediatric medical and surgical subspecialists, and paediatric anesthesiologists.

***Points for discussion:***

- Based on the description given, what is the most appropriate level of neonatal care provided by your facility?
- What suggestions would you like to give in order to improve the current neonatal services to another level? Probe for skills, equipment & other inputs.
- Briefly explain the nature of referral arrangements for new-born care in this facility (for both in-referral and out-referral)? Availability of referral infrastructure (telephone, ambulance and forms)

1. **New born care Provider training and skills**
2. What specific trainings have your staff (nurses/clinical officers/doctors) received in new-born care in the past 3 years? ***Probe*** *for a variety of courses-whether for a normal neonate or handling of neonatal emergencies* or new-born complications*, etc.*
3. In case you have a New Born Unit, are health providers who work in the unit trained? If Yes-probe for the length of time, topics covered, training approaches used e.g. OJT, etc.; practical, follow up, certification
4. What skills and training would be required if the health care providers are to offer comprehensive new born care services in this county?
5. **Equipment, commodities and supply for New born Units**

- Please comment on the equipment for new-born care –focusing on availability and whether they are working/functional.

1. **Heath Management Information System**

Please comment on:

1. Data production /collection and transmission on new-born care services by this HF
2. Timely submission of information and existing feedback mechanisms on new-born care services
3. Dissemination and use of data or information on new-born care services
4. **Financing of New-born services**

Please briefly comment on:

1. Whether there are charges or costs (if any) for providing various new-born care services that are usually borne by the family or households?
2. How does the facility generate revenue or ensure that they have adequate allocation to ensure uninterrupted provision of new-born services?
3. How does the facility handle or protect poor mothers who may not afford to pay for certain new-born care services or investigations?
4. Funding for maintenance of medical equipment including the equipment used for new-born care services (to ensure that they remain functional).
5. **Challenges facing new born care practices**
6. What challenges exist at the community level that may be the main stumbling block to utilization of new born care services in this area or community?
7. What challenges exist within the health facility that may be a barrier to effective utilization of new born care services?
8. How have you been addressing these challenges (both community/facility based ones)?
9. **Sustainability on the future on the new born units**

- Please describe in details what you would like to see of the new-born care services in *five years, and ten years from now.*
- In what ways can the new born care services be made more sustainable?

Finally, we have come to the end of the interview. Is there anything that you would like to add or ask?

**THANK YOU VERY MUCH FOR TAKING YOUR TIME TO TALK TO US**
